# The Phenolic Profile of Sweet Cherry Fruits Influenced by Cultivar/Rootstock Combination

**DOI:** 10.3390/plants12010103

**Published:** 2022-12-25

**Authors:** Djordje Boskov, Dragan Milatovic, Vera Rakonjac, Gordan Zec, Metka Hudina, Robert Veberic, Maja Mikulic-Petkovsek

**Affiliations:** 1 Faculty of Agriculture, University of Belgrade, Nemanjina 6, 11080 Belgrade, Serbia; 2 Department of Agronomy, Biotechnical Faculty, University of Ljubljana, Jamnikarjeva 101, SI-1000 Ljubljana, Slovenia

**Keywords:** phenolic compounds, *Prunus avium* L., rootstock, cultivar, nutritional value

## Abstract

The influence of three cultivars (‘Carmen’, ‘Kordia’ and ‘Regina’) grafted on six rootstocks (Mahaleb, ‘Colt’, ‘Oblacinska’, ‘M × M 14′, ‘Gisela 5′ and ‘Gisela 6′) on the phenolic profile of sweet cherry fruits was studied during a two-year period. All the individual phenolic compounds were detected using high-pressure liquid chromatography with diode-array detection coupled with mass spectrometry (HPLC-DAD-MSn). In all the examined samples, 54 compounds were identified and divided into five phenolic classes: anthocyanins (4 compounds), flavonols (7), flavanols (11), flavanones (4), and hydroxycinnamic acids (28). Anthocyanins (58%) and hydroxycinnamic acids (31%) showed the greatest amounts in all the examined fruit samples. PCA analysis revealed that among the cultivars, ‘Kordia’ showed the highest phenolic content. Regarding rootstocks, the lowest values of the most important phenolic compounds were obtained in fruits from trees grafted onto the seedling rootstock Mahaleb. Among the clonal rootstocks, the vigorous ‘Colt’ and dwarf ‘Gisela 5′ promoted the highest values of the evaluated phenolic compounds in the cultivars ‘Kordia’ and ‘Carmen’, while the dwarf ‘Oblacinska’ and semi-vigorous ‘M × M 14′ induced the highest values in the cultivar ‘Regina’. By evaluating the influence of cultivars and rootstocks on the phenolic content in fruit, it has been proven that the cultivar has the most significant influence. However, the rootstock also influences the content of a large number of phenolic compounds. The selection of an adequate cultivar/rootstock combination can also be a powerful tool for improving the phenolic content in fruits, and consequently the nutritional value of sweet cherry fruits.

## 1. Introduction

In recent decades, sweet cherry production has seen a significant increase and it continues to spread worldwide with the main trends being to improve growing efficacy and ameliorate premium fruit quality [1]. The impact rate is mainly influenced by the production markets of the United States, Chile, and China [2]. With production of over 2.6 million tonnes per year, sweet cherry is in seventh position in the global production of temperate fruits [3].

Sweet cherry fruits are highly valued on the market due to their main sensory attributes such as firmness, sweetness, sourness, and colouration [4]. Fruit quality is not attributed only by appearance, textural and taste properties, but also by the chemical and nutritional compounds in the fruits [5]. Currently, consumer satisfaction is not only based on the primary pleasure of eating these delicious aromatic fruits, but also on their many human health benefits [6,7].

Phenolics are bioactive compounds naturally occurring in plant-derived foods. They are garnering increasing attention from researchers since they have been proven to play a significant role in improving health. [8,9]. The main dietary phenolic compounds play an important role as anti-cancer agents [10]. In the group of phenolics, flavonoids have significant anti-inflammatory and antioxidant benefits, while anthocyanins have a considerable impact on cardiovascular health [11].

Sweet cherries are very nutritious fruits with their proposed health benefits mostly stemming from their high levels of phytochemicals, moderate levels of carbohydrates, and low amounts of calories [10]. Many properties of sweet cherry-based products are associated with the presence and content of their phenolic compounds [12]. The phenolic profile of sweet cherry fruits is mainly determined by the genotype of the cultivar [13,14]. The other important factors include fruit maturity stage [15], orchard management, and storage conditions [11].

Different rootstocks not only influence the efficacy of fruit production and the established modern growing technology, but they also improve fruit quality [16,17]. Worldwide trials have been focused on studying the influence of different rootstocks and cultivars with the purpose of defining the best orchard model, including the most valuated production properties such as yield, vigour, and fruit size [18,19]. Also, it was found that different cultivar/rootstock combinations affect the leaf mineral composition [20], response to water use efficiency [21] and fruit quality [16,22,23].

Several studies have demonstrated the influence of rootstocks on the content of some phenolic compounds in sweet cherry fruits [16,24,25,26]. However, only a few sweet cherry cultivars were considered in these studies. Moreover, the number of phenolic compounds analysed was relatively small. To date, there has been no comprehensive study on the influence of the rootstock on the content of a large number of phenolic compounds in sweet cherry fruit. Also, some cultivars, such as ‘Carmen’, and rootstocks, such as the ‘Oblacinska’ sour cherry and ‘Colt’, have not been studied from this point of view.

The aim of this study was, therefore, to determine the influence of three cultivars and six rootstocks on the phenolic profile of sweet cherries during two growing seasons. The results obtained could help find the best combination of cultivar/rootstock in terms of the content of phenolic compounds as an important indicator of fruit quality.

## 2. Results

### 2.1. The Phenolic Profile Detected in the Sweet Cherry

The phenolic profile detected in the sweet cherry cultivars ‘Carmen’, ‘Kordia’, and ‘Regina’ grafted onto different rootstocks is characterized by 28 compounds, divided into five groups: anthocyanins (4 compounds), flavonols (7), flavanols (5), flavanones (4), and hydroxycinnamic acids (8) (Table 1). The total number of detected and quantified individual phenolic compounds using HPLC-DAD was actually 54. For simple presentation, some individual phenolic compounds are grouped in Table 1, while the complete list of identified compounds is given in the supplementary material (Table S1).

The largest number of identified individual compounds belong to the group of hydroxycinnamic acids (28). Hydroxycinnamic acid derivatives were classified into eight subgroups (caffeoylquinic acid derivatives, coumaroylquinic acid derivatives, caffeic acid derivatives, dicaffeoylquinic acid derivatives, feruloylquinic acid derivatives, ferulic acid derivatives, and *p*-coumaric acid derivatives). Eight of the 11 detected flavanol compounds were classified into two groups (procyanidin dimers and procyanidin trimers). The content of all the detected individual hydroxycinnamic acids and flavanols are presented in the supplementary material (Tables S2 and S3).

### 2.2. Content of Individual and Total Anthocyanins

The amount of the four detected individual anthocyanins (cyanidin-3-rutinoside, cyanidin-3-glucoside, pelargonidin-3-glucoside, and peonidin-3-glucoside), and the content of the total anthocyanins in sweet cherry fruits was significantly influenced by the cultivar, rootstock, the year of study, and the cultivar/rootstock interaction (Table 2).

Cyanidin-3-rutinoside was the most abundant anthocyanin, with content ranging from 128.4 ± 14.3 to 597.1 ± 51.2 mg/kg of fresh weight (FW) followed by cyanidin-3-glucoside, peonidin-3-glucoside, and pelargonidin-3-glucoside, respectively. The combination of the cultivar ‘Kordia’ grafted onto ‘Colt’ rootstocks showed the highest level of the total (663.6 ± 52.8 mg/kg FW) and individual anthocyanin contents, while the combination of the cultivar ‘Regina’ grafted onto Mahaleb rootstocks showed the lowest content of total (141.3 ± 23.7 mg/kg FW) and individual anthocyanins.

The richest genotype with all the individual and total anthocyanins was ‘Kordia’, while the lowest levels were detected in the cultivar ‘Carmen’. There were no significant differences in the content of all the individual anthocyanins between the cultivars ‘Carmen’ and ‘Regina’, while the total anthocyanins were significantly higher in ‘Regina’.

All the individual and total anthocyanin contents were lowest in fruits picked from trees grafted onto ‘Gisela 6′ and Mahaleb rootstocks. On the other hand, the ‘Colt’ rootstock influenced the highest levels of most individual anthocyanin components and in the amount of the total anthocyanins.

Regarding the dominant anthocyanin, cyanidin-3-rutinoside, the highest level was identified in fruits harvested from all the cultivars grafted onto the rootstocks ‘M × M 14′ (448.3 ± 51.2 mg/kg FW) and ‘Colt’ (419.1 ± 60.6 mg/kg FW). The lowest content of cyanidin-3-rutinoside was detected in fruit samples of cultivars grafted onto ‘Gisela 6′ rootstock (207.6 ± 22.9 mg/kg FW) and Mahaleb rootstock (217.7 ± 22.8 mg/kg FW).

Fruits harvested in the year 2020 had a significantly higher level of all individual anthocyanins compared with the samples harvested in the year 2021.

### 2.3. Content of Individual and Total Flavanols

The variability in the content of individual and total flavanols is presented in Table 3. Cultivar, rootstock, and their interaction showed a significant influence on the content of all the individual and total flavanols. However, the influence of the year was not significant for catechin and the total flavanol content.

The level of the total flavanols in sweet cherry fruits in the cultivar/rootstock combinations varied from 29.4 ± 6.3 to 59.2 ± 9.1 mg/kg FW. Among the individual flavanols, procyanidin trimers, procyanidin dimers, and epicatechin had the highest levels. The content of procyanidin trimers ranged from 8.1 ± 0.9 to 22.1 ± 1.7 mg/kg FW, procyanidin dimers from 10.6 ± 1.2 to 21.9 ± 1.9 mg/kg FW, and epicatechin from 4.55 ± 0.98 to 14.04 ± 1.69 mg/kg FW.

The level of the total flavanols was highest in two cultivar/rootstock combinations: ‘Kordia’/’Colt’ (59.1 ± 4.9 mg/kg FW) and ‘Kordia’/’Gisela 5′ (59.2 ± 9.1 mg/kg FW). The lowest content of the total flavanols was found in the combinations of the cultivars ‘Regina’ (29.7 ± 3.5 mg/kg FW) and ‘Carmen’ (29.4 ± 6.3 mg/kg FW) grafted onto Mahaleb rootstock.

Among the rootstocks, Mahaleb influenced the lowest level of the total flavanols (33.4 ± 2.7 mg/kg FW), while the highest amount was induced by the ‘Oblacinska’ rootstock (48.3 ± 3.9 mg/kg FW).

The fruits of the ‘Kordia’ cultivar showed the highest amount of the total flavanol content (44.3 ± 3.5 mg/kg FW), while ‘Carmen’ showed the lowest content (40.3 ± 1.7 mg/kg FW). The contents of catechin, epicatechin, and procyanidin dimers were highest in cultivar ‘Kordia’, while the contents of epicatechin gallate and procyanidin trimers were highest in cultivar ‘Regina’.

### 2.4. Content of Individual and Total Flavonols

The group of flavonol derivatives is presented in Table 4. This phenolic group is composed of the seven individual flavonols (isorhamnetin-3-rutinoside, kaempferol-3-glucoside, kaempferol-3-rutinoside, quercetin-3-galactoside, quercetin-3-glucoside, quercetin-3-rutinoside, and quercetin-7-glucoside-3-rutinoside). The most prominent flavonol in fruits was quercetin-3-rutinoside (rutin). The amount of this individual component varied from 6.53 ± 0.50 to 20.69 ± 2.49 mg/kg FW, which makes 78% percent of all the analysed components in the group of flavonols. Quercetin-7-glucoside-3-rutinoside, quercetin-3-glucoside and quercetin-3-galactoside represented 14.5%, 2.69% and 2.44% of the total flavonols in the examined samples. Isorhamnetin-3-rutinoside, kaempferol-3-glucoside and kaempferol-3-rutinoside participated with less than 1% of the total flavonol content.

The amount of the total flavonols is affected by all the examined factors (cultivar, rootstock, year, and cultivar/rootstock interaction). The content of the total flavonols in samples from the combinations ‘Regina’/Mahaleb (13.6 ± 1.6 mg/kg FW) and ‘Carmen’/Mahaleb (15.1 ± 1.5 mg/kg FW) were half the level of the total flavonol content detected in the combinations ‘Kordia’/’Gisela 5′ (31.0 ± 2.7 mg/kg FW) and ‘Kordia’/’Colt’ (31.9 ± 3.8 mg/kg FW). ‘Kordia’ was the cultivar with the highest, while ‘Carmen’ was the cultivar with the lowest content of the total flavonols. The lowest level of the total flavonols in cherries was on the Mahaleb rootstock (16.2 ± 2.6 mg/kg FW), while significantly higher levels were found on four rootstocks: ‘Gisela 5′, ‘M × M 14′, ‘Oblacinska’, and ‘Colt’ (≥24.1 mg/kg FW).

The content of the main flavanol quercetin-3-rutinoside (rutin) in the sweet cherry fruit samples varied significantly depending on the cultivar/rootstock interaction. The highest amount of quercetin-3-rutinoside was found in sweet cherry samples of the combination ‘Kordia’/’Colt’ (20.69 ± 2.49 mg/kg FW), while only about a third of this content was found in the combinations ‘Carmen’/Mahaleb (7.66 ± 1.37 mg/kg FW) and ‘Regina’/Mahaleb (6.53 ± 0.5 mg/kg FW). Among the cultivars, ‘Kordia’ (17.37 ± 1.20 mg/kg FW) showed the highest level of quercetin-3-rutinoside, while there was no statistically significant difference in rutin content between ‘Carmen’ and ‘Regina’. Among the rootstocks, ‘Colt’ induced the highest content of quercetin-3-rutinoside in sweet cherry fruits, while Mahaleb induced the lowest amount of this flavonol.

For some individual flavonol compounds, significant differences for some experimental factors were not found. For isorhamnetin-3-rutinoside and kaempferol-3-glucoside, differences were not significant between rootstocks and years, for kaempferol-3-rutinoside between cultivars, and for quercetin-3-galactoside between cultivars and years.

### 2.5. Content of Individual and Total Flavanones

Flavanones had the lowest content of all the identified phenolic groups. The content of individual and total flavanones is presented in Table 5. In all the examined samples, four individual flavanones were detected: naringenin hexoside 1, naringenin hexoside 2, taxifolin hexoside, and taxifolin rutinoside. Taxifolin hexoside was the major flavanone detected with an average value of 2.82 mg/kg FW, followed by taxifolin rutinoside (0.93 mg/kg FW), while the contents of naringenin hexoside 1 and naringenin hexoside 2 did not exceed 0.4 mg/kg FW.

Depending on the cultivar, rootstock, their interaction and the year of examination, varying amounts of the total flavanones were found in the cherry fruit samples. The amount of the total flavanones varied between 2.39 ± 017 and 4.65 ± 0.37 mg/kg of FW. The highest level of total flavanones was found in the combinations ‘Carmen’/’Gisela 6′ and ‘Regina’/Mahaleb, while the lowest amount was found in the combination ‘Kordia’/Mahaleb.

The cultivar was also a significant factor affecting the amount of both the total and individual flavanones. ‘Kordia’ influences the highest content of the total flavanones, which was significantly higher than in the cultivar ‘Carmen’. Among the rootstocks, Mahaleb showed the lowest amount of total flavanones (3.20 ± 0.19 mg/kg FW). All other rootstocks influenced the significantly higher level of the total flavanones (≥4.23 mg/kg FW).

### 2.6. Content of Hydroxycinnamic Derivatives and Total Hydroxycinnamic Acids

Hydroxycinnamic acids (HCA) were the most diverse phenolic group found in all the studied samples of three sweet cherry cultivars grafted onto different rootstocks. The content of individual and total hydroxycinnamic acids is presented in Table 6. In the group of HCA, derivatives of caffeoylquinic, caffeic, and coumaroylquinic acid were the most expressed and represent more than 91% of the total hydroxycinnamic acids content. Derivatives of ferulic, sinapic, and dicaffeoylquinic acids were detected in levels lower than 1 mg/kg FW of fruit samples.

The cultivar had a significant influence on the content of the individual and total HCA derivatives. The influence of the rootstock was also significant on all HCA compounds, with the exception of the dicaffeoylquinic acids. The interaction of cultivar/rootstock did not show significant differences between the mean values for three compounds (caffeoylquinic acid derivatives, dicaffeoylquinic acids, and feruloylquinic acid derivatives). Similarly, differences between years were not significant for two compounds (dicaffeoylquinic acids and feruloylquinic acid derivatives).

The content of the total hydroxycinnamic acids in the studied samples varied from 75.9 ± 13.2 to 198.4 ± 17.8 mg/kg FW. The highest level was found in samples of the cultivar ‘Carmen’ grafted onto the dwarf rootstock ‘Colt’ (198.4 ± 17.8 mg/kg FW). The lowest level was found in the cultivar ‘Regina’ grafted onto the vigorous seedling rootstock Mahaleb (75.9 ± 13.2 mg/kg FW).

Caffeoylquinic acid derivatives were the most abundant compounds in this group. Their content ranged from 42.6 ± 4.8 to 87.0 ± 7.5 mg/kg FW. The cultivar ‘Kordia’ had the lowest content, while the cultivars ‘Carmen’ and ‘Regina’ reached a similarly high content of caffeoylquinic acid derivatives. Fruits picked from trees grafted onto the vigorous seedling rootstock Mahaleb had the lowest amount of this compound (44.0 ± 3.4 mg/kg FW), while the dwarf rootstock ‘Gisela 5′ showed the highest amount (69.1 ± 5.5 mg/kg FW). Caffeic acid derivatives varied from 18.4 ± 1.8 to 86.1 ± 9.6 mg/kg FW. The ‘Kordia’/Colt combination expressed the highest (86.1 ± 9.6 mg/kg FW), while the ‘Regina’/Mahaleb combination showed the lowest level (18.4 ± 1.8 mg/kg FW) of caffeic acid derivatives. ‘Regina’ was the cultivar with the lowest amount of this content, while ‘Carmen’ and ‘Kordia’ had a similar, significantly lower level. Regarding rootstock influence, Gisela 6 induced the lowest average content (30.0 ± 2.8 mg/kg FW), while ‘M × M 14′ induced the highest content (65.0 ± 5.6 mg/kg FW).

The amount of coumaroylquinic acid derivatives varied significantly depending on the cultivar/rootstock interaction. The lowest amount of coumaroylquinic acid (10.2 ± 1.4 mg/kg FW) found in the ‘Regina’/Mahaleb combination was 5.7 times lower than in the highest value found in the ‘Carmen’/’Oblacinska’ combination (58.6 ± 3.6 mg/kg FW). The largest amount of coumaroylquinic acid derivatives was found in fruits of the ‘Carmen’ cultivar, while the lowest content was found in samples of the cultivar ‘Regina’. The lowest level of coumaroylquinic derivatives was detected in fruit samples of cultivars grafted onto the vigorous rootstock Mahaleb, while the highest level was found in samples on the dwarf rootstock ‘Gisela 5′.

### 2.7. Principal Component Analysis

**Figure 1:**
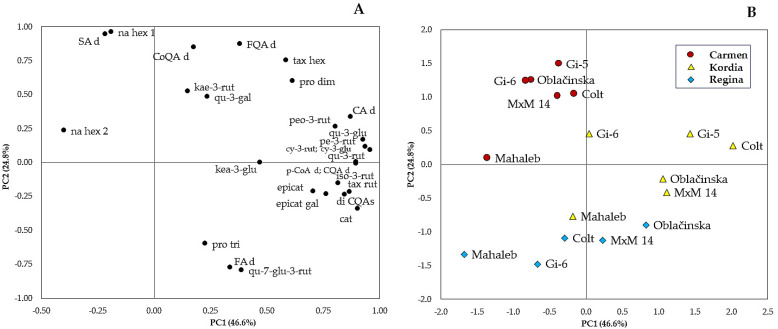
Principal component analysis of loading variables (**A**) and scatter plot (**B**).

Since the content of phenolic compounds varied widely among cultivars, rootstocks, years, and their interaction, a principal component analysis (PCA) was performed in order to provide partial visualization of the dataset in a reduced dimension (Figure 1). PCA produced five PCs with eigenvalues greater than 1, explaining 90.7% of the total variability observed. On the basis of the principal component coefficients between the original variables and these five PCs, using an absolute value greater than 0.75 as a criterion for the significance, it was found that these values are present in the first three PCs. The first principal component contributed 46.6%, the second 24.8%, and the third 11.5% of the total variability obtained.

Component 1 mainly explained the variability in all anthocyanins, quercetin-3-rutinoside, quercetin-3-glucoside, isorhamnetin-3-rutinoside, naringenin hexoside 2, taxifolin hexoside, procyanidin dimers, and derivatives of caffeoylquinic, caffeic, ferulic and *p*-coumaric acids. The second factor (PC2) correlated positively with catechin, taxifolin rutinoside, coumaroylquinic acid derivatives and sinapic acid derivatives and negatively with quercetin-7-glucoside-3-rutinoside and dicaffeoylquinic acids (Figure 1A).

The distribution of cultivar/rootstock combinations along the PC1/PC2 scatter plot (Figure 1B) showed a split into three main groups. The cultivars ‘Regina’ and ‘Carmen’ were negatively linked to the PC1, whereas the cultivar ‘Kordia’ had positive scores for the same component. This arrangement confirms the results of ANOVA, which determined that the cultivar ‘Kordia’ contains significantly more phenolic compounds that are significant and positively correlated within PC1. Discrimination between the cultivars ‘Regina’ and ‘Carmen’ was highlighted on PC2. ‘Carmen’ is located on the positive, and ‘Regina’ is on the negative side of PC2. Furthermore, the distribution of samples within these three main groups indicates a pronounced cultivar/rootstock interaction. In all three cultivars, the worst results in terms of the content of phenolic compounds were obtained from the seedling rootstock Mahaleb. Among the clonal rootstocks, the vigorous ‘Colt’ and dwarf ‘Gisela 5′ promoted the highest values of the evaluated compounds in the cultivars ‘Kordia’ and ‘Carmen’, while the dwarf ‘Oblacinska’ and semi-vigorous ‘M × M 14′ induced the highest values in the cultivar ‘Regina’.

## 3. Discussion

In the three sweet cherry cultivars grafted onto different rootstocks, 54 individual phenolic compounds were detected and quantified. They were classified into five groups: anthocyanins (4 compounds), flavonols (7), flavanols (11), flavanones (4), and hydroxycinnamic acids (28). Anthocyanins accounted for the highest percentage (62.7%) of all analysed phenolics, while hydroxycinnamic accounted for 26.1% of the total phenolic content. Flavanols, flavonols, and flavanones corresponded to 7.2, 3.2, and 0.7% of total phenolics, respectively. The same components were identified in all the samples of cultivar/rootstock combinations studied. Cyanidin-3-rutinoside was the dominant component in the phenolic profile of all the samples studied (on average 43.87% of the total phenolics).

Anthocyanins and hydroxycinnamic acids represent the main phenolic compounds in sweet cherries, as reported previously [27,28]. In all the analysed fruit samples from different cultivars and rootstocks, anthocyanins are the most dominant components. On the other hand, the hydroxycinnamic acids (HCA) represent the most numerous phenolic compounds. This agrees with the previous findings [14,29]. Martini et al. [14] reported 86 tentatively identified phenolics in six different sweet cherry cultivars, of which 40 belong to the class of hydroxycinnamic acids. Gonçalves et al. [29] reported that the phenolic profile of 23 Portuguese sweet cherry cultivars consists of 46 phenolic compounds: 19 hydroxycinnamic acids, 2 hydroxybenzoic acids, 13 flavonols, 5 flavan-3-ols, 2 flavanones, 1 flavanonol and 4 anthocyanins.

The cultivars significantly influenced the total content of all phenolic groups, as well as the content of almost all the detected individual phenolic compounds. The exceptions are only two minor flavanol compounds (kaempferol-3-rutinoside and quercetin-3-galactoside). Our results confirmed the previous finding of a strong genotype influence on the phenolic profile of sweet cherry fruits [29,30,31]. Among the studied cultivars, the highest content of most phenolic compounds was found in ‘Kordia’, then in ‘Regina’, while the lowest content was found in ‘Carmen’. The higher phenolic content in cultivar ‘Kordia’ compared to ‘Regina’ is in agreement with previous research [25].

In a previous study [31], the content of anthocyanins was correlated with the attractiveness of the fresh fruit colour and antioxidant activity. The total anthocyanin content in the cultivar/rootstock combinations ranged from 141.3 to 597.1 mg/kg, which agrees with previous reports [13,32]. The highest percentage of individual anthocyanins had cyanidin-3-rutinoside (90.1%), followed by cyanidin-3-glucoside (6.4%), peonidin-3-rutinoside (2.3%) and pelargonidin-3-rutinoside (1.2%). Our results of individual anthocyanins share are in accordance with previous reports [31,32,33].

Fruits of the cultivar ‘Kordia’ were the richest in major detected anthocyanins, and the values of the total and individual anthocyanins were similar to those found by Milinovic et al. [25] The contents of the total and individual athocyanins were higher than in the ‘Black Star’, ‘Sweetheart’, ‘Sunburst’, ‘Summit’, and ‘Van’ sweet cherry cultivars reported by other authors [34,35]. Mozetič et al. reported a higher amount of cyanidin-3-rutinoside during the late phases of maturation [36], which could be explained by different climatic conditions.

Cyanidin-3-rutinoside and cyanidin-3-glucoside were the main identified anthocyanins, while the derivatives of caffeoylquinic, caffeic, and coumaroylquinic acids were the leading components among the hydroxycinnamic acids in sweet cherry fruits. The rootstock significantly influenced the amount of detected individual and total anthocyanins. Our results confirm previous findings that anthocyanin content was largely affected by the rootstocks in the cultivars ‘Lapins’ [16,24] and ‘0900 Ziraat’ [26].

The influence of the year on some phenolic compounds confirmed the results of the previous research [25,37]. During the first year of examination, the level of anthocyanins and flavanols was considerably higher, while the amount of total flavanones and hydroxycinnamic acids were higher during the second year. The significant effects of the harvest year on phenolic content can be explained by different meteorological factors, such as temperature, solar radiation, and the amount of rain (Table S4). The more than doubled content of all the individual and total anthocyanins detected in the year 2020 can be explained as a response to the weather conditions during the first year of examination. During June 2020, the total precipitation was considerably higher (158 mm) than in 2021 (only 34 mm). Also, the average monthly temperatures during the maturation period (May and June) were considerably higher in the second year of study (2021), especially in June. This was the same case for the time of insolation. The water stress during the ripening period is reported to have a positive effect on the biosynthesis of phenolic compounds in the fruits of apricot [38] and peach [39].

Identification concerning the hydroxycinnamic acid profile is in accordance with previous research, which identifies caffeoylquinic acid derivatives, caffeic acid derivatives, and coumaroylquinic acid derivatives as the major hydroxycinnamic components in cherries [40]. According to the previous results, the content of enumerated components of hydroxycinnamic acids varies greatly depending on the cultivar and rootstock [23,25,41]. The dominance of caffeoylquinic acid derivatives in all the samples was the same as in the plum cultivar ‘Čačanska lepotica’ grafted onto five different rootstocks [42].

The most prominent flavonols detected in our study were quercetin-3-rutinoside and quarcetin-7-glucoside-3-rutinoside. Our values of flavonol glycosides are in accordance with previous studies [28]. It was also found earlier that quercetin-3-rutinoside is the most dominant compound of flavonols and that it is significantly influenced by the rootstock [24].

The detected individual flavanols were reported to be the same as components detected in six ancient sweet cherry cultivars [12]. The obtained results of epicatechin content were in agreement with the report of Kelebek et al. [43] concerning different cultivars, while the content of catechin was half of what was detected in our report. Mikulic-Petkovsek et al. detected higher amounts of procyanidin dimers and procyanidin trimers in wild *Prunus* species [44] than the values found in our study. It is worth mentioning that the influence of cultivar/rootstock interaction on procyanidin dimers and trimers has not been reported so far.

Flavanones are determined as the smaller group of the phenolic profile representing 1% of the total phenolic compounds. They included four compounds that are in all the examined samples. Two of them are naringenin hexosides, which confirms the results of Gonçalves et al. [29]. The same low content of naringenins was found in raw and frozen sweet cherry fruits, as well as in cherry juice [45].

By PCA, out of the 28 phenolic compounds detected in sweet cherry fruits, 22 showed strong correlations with PCs, namely 14 with PC1, 7 with PC2, and one with PC3 which indicates the high discriminating power of these compounds. These results agree with previous reports, which indicate that it is possible to differentiate between sweet cherry genotypes using PCA based on their phenolic constituents [14,30,34,37,46].

The classification of different cultivar/rootstock combinations into three main groups is primarily a function of the genetic potential of the cultivar. This is in agreement with the results of Radović et al. [22] who studied the chemical composition of three plum cultivars grafted onto four rootstocks and concluded that the chemical composition of the fruits was more cultivar-than rootstock-dependent. In general, the highest phenolic content was found in the cultivar ‘Kordia’, followed by ‘Carmen’ and ‘Regina’.

The highest variation in phenolic amount due to the rootstock was found in the cultivar ‘Kordia’, slightly less in the cultivar ‘Regina’, while the cultivar ‘Carmen’ showed the greatest stability concerning the phenolic content on different rootstocks. In all three cultivars, the lowest content of phenolic compounds was obtained in fruits from the vigorous seedling rootstock Mahaleb. Among the clonal rootstocks, there was not clear influence of vigour on the content of phenolic compounds in cherry fruit. In the cultivars ‘Kordia’ and ‘Carmen’, semi-vigorous ‘Colt’ and dwarf ‘Gisela 5′ promoted the highest values of phenolic compounds, while in the cultivar ‘Regina’ the semi-vigorous ‘M × M 14′ and the dwarf ‘Oblacinska’ induced the highest values. The results obtained in our research show that rootstock vigour is not linearly correlated with the content of phenolic compounds in the cherry fruit, which is consistent with data shown by Remorini et al. [47] who reported that the phenolic levels in peaches were influenced by the rootstock, but its vigour did not affect some secondary metabolites. Similarly, Milošević et al. [48] cited that one dwarf (low vigorous) and one vigorous rootstock promoted the best values of the evaluated compounds and antioxidant capacity in the sour cherry cultivar ‘Šumadinka’. Jakobek et al. [27] considered that higher phenolic compound content in sweet cherry fruits probably comes from heterogenic grafting combinations. The differences in the concentration of bioactive compounds in sweet cherry fruit are explained by the effects of the rootstocks on scion physiology [49]. Karakaya et al. [26] found that incompatibility problems can affect the content of individual phenolic compounds in sweet cherry fruits.

## 4. Materials and Methods

### 4.1. Plant Material

The sweet cherry fruits were collected in two consecutive years (2020 and 2021) from the 7- and 8-year-old experimental plantation located at the Fruit Growing Centre "Radmilovac" of the Faculty of Agriculture in Belgrade (44°75′ N 20°58′ E; 110 m altitude). The experimental design includes fruits of the cultivars ‘Carmen’ and ‘Kordia’ grafted onto six different rootstocks (Mahaleb, ‘Colt’, ‘Oblacinska’, ‘M × M 14′, ‘Gisela 5′, and ‘Gisela 6′), while the cultivar ‘Regina’ was grafted onto five different rootstocks (Mahaleb, ‘Colt’, ‘Oblacinska’, ‘M × M 14′, and ‘Gisela 6′). ‘Carmen’ cultivar originates from Hungary and is low to moderate in terms of vigour and has a mid-early ripening time. Cultivars ‘Kordia’ (from Czech Republic) and ‘Regina’ (from Germany) have a late time ripening time and high vigorous trees. Mahaleb (*Prunus mahaleb*) is the only seedling rootstock. Other rootstocks are clonal and have a different origin. ‘Oblacinska’ is a sour cherry (*Prunus cerasus*), while other rootstocks have a hybrid origin: *P. avium* × *P. pseudocerasus* (‘Colt’), *P. mahaleb* × *P. avium* (‘M × M 14′), and *P. cerasus* × *P. canescens* (‘Gisela 5′ and ‘Gisela 6′). Regarding the rootstock vigour, Mahaleb and ‘Colt’ have high vigour, ‘M × M 14′ is semi-vigorous, ‘Gisela 6′ is semi-dwarfing (low vigour), while ‘Oblacinska’ and ‘Gisela 5′ are dwarfing (very low vigour). Ten trees of every cultivar/rootstock combination were planted. For each combination, three replicates were carried out (n = 3); each replicate included 25 fruits per combination. Fruits were picked at the commercial maturity stage based on earlier experience for each cultivar, with maturity indicators such as firmness, colour, and soluble solids content being used. The planting distance in the orchard is designed in accordance with the rootstock vigour. The distance between rows is 4 m, while within rows it is 3 m (Mahaleb and ‘Colt’), 2.5 m (‘M × M 14′), 2.2 m (‘Gisela 6′), and 1.7 m (‘Oblacinska’ and ‘Gisela 5′). The spindle training system was used for all cultivar/rootstock combinations. The fruit samples were frozen and kept in the freezer at a temperature of −18 ℃.

### 4.2. Extraction and Analysis of Phenolic Compounds

Extraction and identification of phenolic compounds in cherries were performed as previously described by Mikulic-Petkovsek et al. [44]. Each cherry sample for extraction of individual phenolic components was weighed with 4 g of mixed fresh fruit, to which 10 mL of extraction solution (methanol/water/formic acid = 70/27/3, v/v/v) was added. Then, the extraction of phenolics was carried out in an ultrasonic bath for 60 min. After that, all extracts were centrifuged at 9000 rpm and the supernatant was filtered through PTFE filters (Macheery Nagel) into vials. Thermo Dionex HPLC system (Thermo Scientific, San Jose, USA) was used in conjunction with a diode array detector (DAD) for the determination of phenolic compounds. The analytical HPLC conditions were the same as previously described by Mikulic-Petkovsek et al. [50]. Phenomenex column (150 × 4.6 mm i.d., 3 μm, Gemini C18) heated to 25 °C was used for separation of phenolic compounds. The studied extract was injected at 20 μL, and the flow rate of the mobile phases was 0.6 mL per minute. The mobile phases were aqueous 0.1% formic acid and 3% acetonitrile in double-distilled water (A), and 0.1% formic acid and 3% distilled water dissolved in acetonitrile (B). Mixing of the mobile phases was performed according to the gradient method described in the study by Mikulic-Petkovsek et al. [51].

Xcalibur software (Thermo Scientific, CA, USA) was used to analyse the spectral data. Identification of phenolic compounds was based on the retention times and PDA spectra of the compounds compared to those of standard phenolic compounds and on the fragmentation patterns in different MSn modes (LTQ XL Linear Ion Trap Mass Spectrometer, Thermo Fisher Scientific, Waltham, MA, USA) compared to published data. All parameters established for mass spectrometry were the same as previously reported by Mikulic-Petkovsek et al. [44]. Using the standard curves of the different phenolic compounds, the concentration of the specific phenolic compounds was calculated. Five different concentrations of each phenolic compound were injected three times to generate standard curves. The phenolic contents were expressed in mg/kg of fresh weight (FW). Due to the identical chemical structure and more compact presentation, some individual compounds are summarised: procyanidin dimmers (procyanidin dimer 1, procyanidin dimer 2, procyanidin dimer 3, procyanidin dimer 4, procyanidin dimer 5 and procyanidin dimer 6), procyanidin trimers (procyanidin trimer 1 and procyanidin trimer 2), caffeoylquinic acid derivatives (caffeoylquinic acid glycoside 1, caffeoylquinic acid glycoside 2, caffeoylquinic acid glycoside 3, *cis*-3-caffeoylquinic acid, *trans* 4-caffeoylquinic acid, *trans* 5-caffeoylquinic acid, *cis* 4-caffeoylquinic acid, *cis* 5-caffeoylquinic acid and neochlorogenic acid), coumaroylquinic acid derivatives (3-*p*-coumaroylquinic acid, 4-*p*-coumaroylquinic acid, *cis*-3-p-coumaroylquinic acid, *trans* 4-*p*-coumaroylquinic acid, *cis*-4-*p*-coumaroylquinic acid and 5-*p*-coumaroylquinic acid), caffeic acid derivatives (caffeic acid glycoside 1, caffeic acid glycoside 2, caffeic acid hexoside 1 and caffeic acid hexoside 2), dicaffeoylquinic acids (dicaffeoylquinic acid 1 and dicaffeoylquinic acid 2), feruoylquinic acid derivatives (*cis* 3-feruloyquinic acid, *trans* 3-feruloyquinic acid, *trans*-5-feruloylquinic acid and feruloyl hexoside), sinapic acid derivatives (sinapic acid hexoside), ferulic acid derivatives (feruloyl hexoside), *p*-coumaric acid derivatives (*p*-coumaric acid hexoside). The total content of phenolic compounds in each class was calculated as a sum of individual components.

### 4.3. Statistical Analysis

All statistical analyses were performed using the “Statistica” (Stat Soft software Inc., Tulsa, OK, USA) program package. Three-way ANOVA was used for the analysis of the effect of cultivar, rootstocks, year, and cultivar/rootstock interaction. Differences between the mean values were estimated with Tukey’s test (*p* < 0.05). Multivariate statistical analysis was conducted in order to interpret the differences between the phenolic compounds in fruits of the cultivars on different rootstocks. For every compound, mean values and standard errors are presented (mean ± SE) and statistical differences among treatments are denoted by different letters.

## 5. Conclusions

Evaluation of the results of all the identified individual phenolic compounds in the three sweet cherry cultivars grafted onto six rootstocks revealed that the phenolic content depends mainly on the cultivar, but is also modified by the rootstock, the interaction between the cultivar/rootstock, and the weather conditions during the study years. The dominant phenolic components were cyanidin-3-rutinoside, caffeoylquinic acid derivatives, and caffeic acid derivatives. The highest amounts of the most phenolic compounds were found in fruits of the cultivar ‘Kordia’. The significant variability of phenolic compounds was also caused by the rootstock. The seedling rootstock Mahaleb had the lowest content of phenolic compounds. Among the clonal rootstocks, semi-vigorous ‘Colt’ and dwarf ‘Gisela 5′ induced the highest values of major phenolic compounds in the cultivars ‘Kordia’ and ‘Carmen’, while the dwarfing rootstock ‘Oblacinska’, and semi-dwarfing rootstock ‘M × M 14′ induced the highest values in the cultivar ‘Regina’. As for the phenolic profile, the best-evaluated cultivar and rootstock combination was ‘Kordia’ grafted onto the ‘Colt’ rootstock. In summary, we can conclude that the phenolic content of sweet cherries, as an important element of fruit quality, can be improved not only by the choice of cultivars and rootstocks, but also by the selection of the best combination of cultivar and rootstock. Thus, the selection of an appropriate combination of cultivar and rootstock is an effective tool for improving the nutritional value of sweet cherries.

## Figures and Tables

**Table 1:** Phenolic compounds detected in sweet cherry fruit and used abbreviations.

Anthocyanins		Flavanones	
Cyanidin-3-rutinoside	cy-3-rut	Naringenin hexoside 1	na hex 1
Cyanidin-3-glucoside	cy-3-glu	Naringenin hexoside 2	na hex 2
Pelargonidin-3-rutinoside	pe-3-rut	taxifolin rutinoside	tax rut
Peonidin-3-rutinoside	peo-3-rut	taxifolin hexoside	tax hex
**Flavonols**		**Hydroxycinnamic acids**	
Quercetin-7-glucoside-3-rutinoside)	qu-7-glu-3-rut	Caffeoylquinic acid derivatives	CQA d
Quercetin-3-rutinoside	qu-3-rut	Coumaroylquinic acid derivatives	CoQA d
Quercetin-3-galactoside	qu-3-gal	Caffeic acid derivatives	CAd
Quercetin-3-glucoside	qu-3-glu	Dicaffeoylquinic acids	di CQAs
Kaempferol-3-rutinoside	kae-3-rut	Feruloylquinic acid derivatives	FQA d
Isorhamnetin-3-rutinoside	iso-3-rut	Sinapic acid derivatives	SA d
Kaempferol-3-glucoside	kae-3-glu	Ferulic acid derivatives	FA d
**Flavanols**		*p*-coumaric acid derivatives	p-CoA d
Procyanidin dimers	pro dim		
Catechin	cat		
Epicatechin	epicat		
Procyanidin trimers	pro tri		
Epicatechin gallate	epicat gal		

**Table 2:** Content of individual and total anthocyanins in fruits of sweet cherry cultivars grafted on different rootstocks (mg/kg FW, average 2020–2021).

Combination Cultivar/Rootstock		Cyanidin-3- rutinoside	Cyanidin-3- glucoside	Pelargonidin-3- glucoside	Peonidin-3- glucoside	Total Anthocyanins
Carmen/Mahaleb		208.8 ± 32.6 ^g–h^	14.7 ± 2.3 ^e–h^	2.2 ± 0.2 ^d–e^	4.3 ± 0.9 ^de^	229.2 ± 38.6 ^f–g^
Carmen/Colt		358.1 ± 36.9 ^b–g^	25.2 ± 2.6 ^b–g^	4.8 ± 0.6 ^b–d^	8.8 ± 1.0 ^b–d^	396.9 ± 31.7 ^b–e^
Carmen/Oblacinska		261.4 ± 23.4 ^g–h^	18.4 ± 1.5 ^d–h^	3.1 ± 0.8 ^c–e^	6.6 ± 1.3 ^c–e^	289.5 ± 43.1 ^c–g^
Carmen/M × M 14		345.9 ± 38.3 ^b–g^	24.3 ± 2.7 ^b–g^	3.7 ± 0.7 ^c–e^	10.8 ± 1.7 ^a–c^	384.7 ± 68.9 ^b–e^
Carmen/Gisela 5		303.3 ± 36.7 ^c–h^	21.3 ± 2.7 ^c–h^	3.9 ± 1.0 ^c–e^	7.7 ± 2.5 ^c–e^	336.2 ± 58.7 ^b–f^
Carmen/Gisela 6		202.8 ± 28.6 ^f–h^	14.3 ± 2.0 ^f–h^	2.5 ± 0.5 ^d–e^	3.6 ± 1.1 ^de^	223.2 ± 28.3 ^g–i^
Kordia/Mahaleb		315.9 ± 21.9 ^c–h^	22.2 ± 1.5 ^c–h^	3.5 ± 0.5 ^c–e^	7.1 ± 1.1 ^c–e^	342.1 ± 46.6 ^e–g^
Kordia/Colt		597.1 ± 51.2 ^a^	42.0 ± 2.6 ^a^	8.6 ± 1.1 ^a^	15.9 ± 3.0 ^a^	663.6 ± 52.8 ^a^
Kordia/Oblacinska		400.4 ± 42.4 ^a–f^	28.2 ± 2.1 ^a–f^	5.7 ± 0.8 ^a–c^	8.3 ± 2.5 ^cd^	442.6 ± 39.7 ^b–f^
Kordia/M × M 14		537.3 ± 64.5 ^ab^	37.8 ± 2.5 ^ab^	5.7 ± 0.9 ^a–c^	14.2 ± 2.2 ^ab^	595.0 ± 51.8 ^ab^
Kordia/Gisela 5		406.1 ± 52.9 ^a–e^	28.6 ± 3.5 ^a–f^	7.6 ± 1.2 ^ab^	15.5 ± 3.1 ^a^	457.8 ± 56.2 ^b–e^
Kordia/Gisela 6		253.6 ± 37.2 ^g–h^	17.8 ± 2.6 ^e–h^	2.6 ± 0.5 ^c–e^	3.7 ± 1.1 ^de^	266.5 ± 32.6 ^e–g^
Regina/Mahaleb		128.4 ± 14.3 ^h^	9.0 ± 1.0 ^h^	1.5 ± 0.2 ^e^	2.4 ± 0.3 ^e^	141.3 ± 23.7 ^i^
Regina/Colt		302.3 ± 38.6 ^c–h^	21.3 ± 2.1 ^c–h^	3.8 ± 0.6 ^c–e^	5.4 ± 0.9 ^c–e^	332.8 ± 49.4 ^d–g^
Regina/Oblacinska		468.2 ± 38.4 ^a–c^	32.9 ± 2.5 ^a–d^	4.8 ± 0.3 ^b–d^	6.5 ± 0.4 ^c–e^	512.4 ± 53.6 ^a–c^
Regina/M × M 14		461.6 ± 32.0 ^a–d^	32.5 ± 3.3 ^a–d^	4.2 ± 0.9 ^c–e^	6.6 ± 2.4 ^c–e^	504.9 ± 51.1 ^a–d^
Regina/Gisela 6		161.5 ± 22.3 ^gh^	11.2 ± 1.3 ^gh^	2.4 ± 0.3 ^d–e^	3.4 ± 0.5 ^de^	178.5 ± 22.8 ^hi^
Cultivar	Carmen	280.1 ± 20.5 ^b^	19.7 ± 1.4 ^b^	3.4 ± 0.3 ^b^	7.0 ± 0.7 ^bc^	303.2 ± 23.8 ^c^
	Kordia	418.4 ± 31.7 ^a^	29.4 ± 2.6 ^a^	5.6 ± 0.8 ^a^	10.8 ± 1.4 ^a^	464.2 ± 32.3 ^a^
	Regina	304.4 ± 40.8 ^b^	21.4 ± 2.9 ^b^	3.4 ± 0.3 ^b^	4.9 ± 0.6 ^c^	334.1 ± 33.7 ^b^
Rootstock	Mahaleb	217.7 ± 22.8 ^c^	15.3 ± 1.6 ^cd^	2.4 ± 0.3 ^b^	4.6 ± 0.7 ^cd^	240.1 ± 20.9 ^c^
	Colt	419.1 ± 60.6 ^a^	29.5 ± 3.3 ^a^	5.7 ± 1.1 ^a^	10.0 ± 1.9 ^a^	464.3 ± 46.7 ^a^
	Oblacinska	376.7 ± 44.1 ^ab^	26.5 ± 3.1 ^ab^	4.6 ± 0.7 ^a^	7.2 ± 0.9 ^bc^	416.5 ± 39.0 ^b^
	M × M 14	448.3 ± 51.2 ^a^	31.5 ± 3.6 ^a^	4.5 ± 0.5 ^a^	10.6 ± 1.4 ^a^	494.9 ± 42.3 ^a^
	Gisela 5	304.1 ± 42.0 ^bc^	21.4 ± 3.0 ^bc^	4.6 ± 0.9 ^a^	9.0 ± 2.4 ^b^	339.1 ± 36.3 ^b^
	Gisela 6	207.6 ± 22.9 ^c^	14.5 ± 1.7 ^d^	2.5 ± 0.3 ^b^	3.6 ± 0.6 ^d^	228.2 ± 22.6 ^c^
Year	2020	393.74 ± 38.69 ^a^	27.68 ± 2.72 ^a^	4.65 ± 0.59 ^a^	8.47 ± 1.04 ^a^	434.5 ± 44.01 ^a^
	2021	273.39 ± 14.81 ^b^	19.24 ± 1.04 ^b^	3.25 ± 0.2 ^b^	5.92 ± 0.58 ^b^	301.8 ± 18.24 ^b^
Statistical significance						
Cultivar		***	***	***	***	***
Rootstock		***	***	***	***	***
Year		***	***	***	***	***
Cultivar × Rootstock		***	***	*	**	***

Data are presented as means ± standard errors (*n* = 3). Different superscript letter in a same column (factor) denotes significant difference (Tukey’s test, *p* < 0.05). Statistical significance: * *p* < 0.05; ** *p* < 0.01; *** *p* < 0.001.

**Table 3:** Content of individual and total flavanols in fruits of sweet cherry cultivars grafted on different rootstocks (mg/kg FW, average 2020–2021).

Combination Cultivar/Rootstock		Catechin	Epicatechin	Epicatechin Gallate	Procyanidin Dimers	Procyanidin Trimers	Total Flavanols
Carmen/Mahaleb		2.13 ± 0.28 ^c–f^	7.87 ± 0.84 ^b–d^	0.7 5± 0.21 ^f^	10.6 ± 1.2 ^f^	8.1 ± 0.9 ^f^	29.4 ± 6.3 ^e^
Carmen/Colt		2.67 ± 0.12 ^a–e^	10.65 ± 0.94 ^a–c^	0.90 ± 0.23 ^f^	13.1 ± 0.9 ^c–f^	7.1 ± 0.3 ^f^	34.4 ± 6.5 ^de^
Carmen/Oblacinska		3.14 ± 0.16 ^a–c^	11.83 ± 0.71 ^ab^	2.28 ± 0.33 ^b–d^	12.9 ± 1.2 ^c–f^	12.5 ± 1.7 ^de^	40.2 ± 3.9 ^b–e^
Carmen/M × M 14		2.49 ± 0.12 ^a–f^	11.34 ± 0.72 ^ab^	0.67 ± 0.14 ^f^	13.7 ± 0.8 ^c–f^	8.3 ± 0.5 ^f^	36.5 ± 7.9 ^c–e^
Carmen/Gisela 5		3.04 ± 0.29 ^a–d^	10.19 ± 1.24 ^a–c^	1.30 ± 0.28 ^d–f^	14.7 ± 2.0 ^b–f^	9.9 ± 0.9 ^ef^	39.1 ± 3.4 ^b–e^
Carmen/Gisela 6		2.58 ± 0.24 ^a–f^	8.90 ± 0.48 ^a–d^	1.15 ± 0.20 ^ef^	11.2 ± 0.7 ^ef^	9.6 ± 1.3 ^ef^	33.4 ± 7.4 ^de^
Kordia/Mahaleb		1.93 ± 0.07 ^d–f^	8.10 ± 1.04 ^b–d^	1.68 ± 0.31 ^c–f^	14.9 ± 1.3 ^b–f^	14.5 ± 0.8 ^b–d^	41.1 ± 4.3 ^b–d^
Kordia/Colt		3.63 ± 0.27 ^a^	14.04 ± 1.69 ^a^	1.63 ± 0.28 ^c–f^	21.9 ± 1.9 ^a^	17.9 ± 1.3 ^bc^	59.1 ± 4.9 ^a^
Kordia/Oblacinska		2.99 ± 0.29 ^a–d^	9.13 ± 1.16 ^a–d^	2.33 ± 0.20 ^b–d^	18.1 ± 1.4 ^a–c^	17.9 ± 1.5 ^bc^	50.4 ± 5.9 ^ab^
Kordia/M × M 14		2.31 ± 0.38 ^b–f^	8.85 ± 0.79 ^a–d^	1.44 ± 0.21 ^c–f^	19.3 ± 1.5 ^ab^	13.3 ± 1.2 ^de^	45.2 ± 6.1 ^bc^
Kordia/Gisela 5		3.43 ± 0.17 ^ab^	11.88 ± 1.84 ^ab^	2.45 ± 0.19 ^a–c^	19.3 ± 1.4 ^ab^	22.1 ± 1.7 ^a^	59.2 ± 9.1 ^a^
Kordia/Gisela 6		3.18 ± 0.33 ^a–c^	10.57 ± 1.32 ^a–c^	2.02 ± 0.18 ^b–e^	15.8 ± 1.4 ^b–f^	18.3 ± 0.6 ^ab^	49.9 ± 5.8 ^ab^
Regina/Mahaleb		0.71 ± 0.07 ^g^	4.55 ± 0.98 ^d^	1.97 ± 0.31 ^b–e^	12.4 ± 0.6 ^d–f^	10.1 ± 1.0 ^ef^	29.7 ± 3.5 ^e^
Regina/Colt		1.70 ± 0.35 ^e–g^	9.55 ± 1.57 ^a–d^	2.04 ± 0.29 ^b–e^	16.4 ± 1.9 ^b–e^	13.2 ± 1.4 ^de^	42.9 ± 5.9 ^b–d^
Regina/Oblacinska		2.29 ± 0.33 ^b–f^	11.73 ± 1.08 ^ab^	3.00 ± 0.27 ^ab^	19.4 ± 0.6 ^ab^	14.1 ± 1.4 ^cd^	50.5 ± 5.4 ^ab^
Regina/M × M 14		1.60 ± 0.38 ^e–g^	7.30 ± 1.06 ^b–d^	2.47 ± 0.25 ^a–c^	17.9 ± 1.4 ^a–c^	12.7 ± 1.6 ^de^	41.9 ± 6.7 ^b–d^
Regina/Gisela 6		1.44 ± 0.13 ^fg^	5.46 ± 0.61 ^cd^	3.48 ± 0.13 ^a^	16.9 ± 0.6 ^a–d^	14.3 ± 0.8 ^cd^	41.6 ± 4.5 ^b–d^
Cultivar	Carmen	2.67 ± 0.11 ^a^	10.13 ± 0.62 ^ab^	1.17 ± 0.13 ^c^	12.7 ± 0.6 ^b^	13.7 ± 1.2 ^ab^	40.3 ± 1.7 ^b^
	Kordia	2.97 ± 0.23 ^a^	10.43 ± 0.73 ^a^	1.93 ± 0.11 ^b^	18.2 ± 0.8 ^a^	10.5 ± 0.5 ^b^	44.3 ± 3.5 ^a^
	Regina	1.55 ± 0.15 ^b^	7.72 ± 0.75 ^b^	2.59 ± 0.17 ^a^	16.6 ± 0.6 ^ab^	15.8 ± 0.7 ^a^	44.1 ± 2.6 ^a^
Rootstock	Mahaleb	1.59 ± 0.18 ^c^	6.84 ± 0.85 ^b^	1.47 ± 0.21 ^b^	12.6 ± 0.7 ^b^	10.9 ± 0.8 ^d^	33.4 ± 2.7 ^c^
	Colt	2.67 ± 0.33 ^a^	11.41 ± 1.64 ^a^	1.52 ± 0.22 ^b^	17.1 ± 1.4 ^a^	12.7 ± 1.7 ^cd^	45.4 ± 5.8 ^ab^
	Oblacinska	2.81 ± 0.17 ^a^	10.90 ± 0.97 ^a^	2.54 ± 0.17 ^a^	16.8 ± 1.1 ^a^	14.9 ± 1.4 ^ab^	48.3 ± 3.9 ^a^
	M × M 14	2.13 ± 0.19 ^bc^	9.17 ± 0.58 ^ab^	1.52 ± 0.21 ^b^	17.0 ± 0.9 ^a^	11.4 ± 0.8 ^d^	41.2 ± 4.3 ^bc^
	Gisela 5	2.82 ± 0.18 ^a^	10.89 ± 1.21 ^a^	1.77 ± 0.19 ^b^	17.0 ± 1.4 ^a^	16.0 ± 1.6 ^a^	46.5 ± 6.0 ^ab^
	Gisela 6	2.60 ± 0.20 ^ab^	7.15 ± 0.65 ^b^	2.54 ± 0.30 ^a^	14.6 ± 0.8 ^ab^	14.1 ± 1.0 ^bc^	41.0 ± 4.3 ^bc^
Year	2020	2.46 ± 0.16	11.98 ± 0.69 ^a^	1.73 ± 0.13 ^b^	15.2 ± 1.9 ^b^	12.4 ± 1.6 ^b^	43.8 ± 2.5
	2021	2.39 ± 0.14	7.07 ± 0.57 ^b^	1.98 ± 0.14 ^a^	16.4 ± 1.0 ^a^	14.0 ± 1.3 ^a^	41.8 ± 1.6
Statistical significance							
Cultivar		***	***	***	***	***	***
Rootstock		***	***	***	***	***	***
Year		ns	***	*	*	***	ns
Cultivar × Rootstock		*	***	**	*	**	*

Data are presented as means ± standard errors (*n* = 3). Different superscript letter in a same column (factor) denotes significant difference (Tukey’s test, *p* < 0.05). Statistical significance: ns—not significant; * *p* < 0.05; ** *p* < 0.01; *** *p* < 0.001.

**Table 4:** Content of individual and total flavonols in fruits of sweet cherry cultivars grafted on different rootstocks (mg/kg FW, average 2020–2021).

Combination Cultivar/Rootstock		Isorhamnetin -3-rutinoside	Kaempferol -3-glucoside	Kaempferol -3-rutinoside	Quercetin-3- galactoside	Quercetin-3- glucoside	Quercetin-3- rutinoside	Quercetin-7- glucoside-3- rutinoside	Total Flavonols
Carmen/Mahaleb		0.03 ± 0.006 ^d^	0.06 ± 0.02 ^c^	0.07 ± 0.02 ^c^	0.33 ± 0.03 ^b–d^	0.25 ± 0.05 ^d^	7.66 ± 1.37 ^d^	0.96 ± 0.28 ^c^	15.1 ± 1.5 ^d^
Carmen/Colt		0.03 ± 0.013 ^d^	0.13 ± 0.02 ^a–c^	0.26 ± 0.05 ^ab^	0.49 ± 0.08 ^a–d^	0.58 ± 0.19 ^a–d^	16.21 ± 1.70 ^a–d^	0.98 ± 0.15 ^c^	29.0 ± 2.8 ^a–c^
Carmen/Oblacinska		0.02 ± 0.007 ^d^	0.11 ± 0.03 ^a–c^	0.10 ± 0.03 ^bc^	0.42 ± 0.05 ^a–d^	0.37 ± 0.09 ^cd^	9.84 ± 0.70 ^cd^	1.52 ± 0.19 ^c^	20.9 ± 2.3 ^b–d^
Carmen/M × M 14		0.03 ± 0.009 ^d^	0.06 ± 0.01 ^c^	0.11 ± 0.04 ^bc^	0.37 ± 0.07 ^b–d^	0.36 ± 0.14 ^cd^	11.97 ± 1.42 ^b–d^	1.04 ± 0.38 ^c^	22.9 ± 2.7 ^a–d^
Carmen/Gisela 5		0.03 ± 0.008 ^d^	0.11 ± 0.01 ^a–c^	0.22 ± 0.05 ^a–c^	0.44 ± 0.06 ^a–d^	0.40 ± 0.12 ^cd^	11.90 ± 1.06 ^b–d^	1.28 ± 0.28 ^c^	22.9 ± 2.4 ^a–d^
Carmen/Gisela 6		0.03 ± 0.010 ^d^	0.16 ± 0.02 ^a–c^	0.36 ± 0.03 ^a^	0.77 ± 0.24 ^a^	0.40 ± 0.08 ^cd^	12.94 ± 1.21 ^a–d^	1.13 ± 0.19 ^c^	20.1 ± 1.9 ^b–d^
Kordia/Mahaleb		0.06 ± 0.009 ^a–d^	0.07 ± 0.01 ^bc^	0.10 ± 0.04 ^bc^	0.41 ± 0.11 ^a–d^	0.43 ± 0.07 ^b–d^	12.18 ± 1.87 ^b–d^	2.90 ± 0.18 ^b^	20.2 ± 3.3 ^b–d^
Kordia/Colt		0.08 ± 0.013 ^a^	0.13 ± 0.04 ^a–c^	0.16 ± 0.03 ^bc^	0.43 ± 0.06 ^a–d^	0.85 ± 0.05 ^ab^	20.69 ± 2.49 ^a^	2.89 ± 0.34 ^b^	31.9 ± 3.8 ^a^
Kordia/Oblacinska		0.08 ± 0.022 ^a^	0.21 ± 0.03 ^a^	0.22 ± 0.08 ^a–c^	0.41 ± 0.08 ^a–d^	0.75 ± 0.12 ^a–c^	17.64 ± 1.68 ^a–c^	2.67 ± 0.22 ^b^	28.1 ± 2.3 ^a–c^
Kordia/M × M 14		0.07 ± 0.007 ^ab^	0.10 ± 0.01 ^a–c^	0.10 ± 0.01 ^bc^	0.37 ± 0.05 ^b–d^	0.73 ± 0.09 ^a–c^	19.47 ± 1.81 ^ab^	2.96 ± 0.23 ^b^	29.0 ± 2.2 ^ab^
Kordia/Gisela 5		0.07 ± 0.019 ^a–c^	0.19 ± 0.03 ^ab^	0.23 ± 0.03 ^a–c^	0.70 ± 0.19 ^ab^	0.95 ± 0.16 ^a^	19.78 ± 1.79 ^ab^	3.30 ± 0.38 ^b^	31.0 ± 2.7 ^a^
Kordia/Gisela 6		0.05 ± 0.006 ^a–d^	0.11 ± 0.04 ^a–c^	0.17 ± 0.02 ^bc^	0.66 ± 0.17 ^a–c^	0.56 ± 0.09 ^a–d^	14.42 ± 1.84 ^a–d^	3.10 ± 0.38 ^b^	28.8 ± 2.5 ^a–d^
Regina/Mahaleb		0.03 ± 0.003 ^d^	0.08 ± 0.02 ^bc^	0.07 ± 0.04 ^c^	0.36 ± 0.05 ^b–d^	0.21 ± 0.07 ^d^	6.53 ± 0.50 ^d^	2.84 ± 0.36 ^b^	13.6 ± 1.6 ^d^
Regina/Colt		0.03 ± 0.005 ^d^	0.10 ± 0.01 ^a–c^	0.08 ± 0.01 ^c^	0.32 ± 0.03 ^cd^	0.35 ± 0.03 ^cd^	12.20 ± 1.34 ^b–d^	3.16 ± 0.49 ^b^	20.1 ± 2.7 ^b–d^
Regina/Oblacinska		0.03 ± 0.003 ^d^	0.12 ± 0.01 ^a–c^	0.14 ± 0.03 ^bc^	0.54 ± 0.11 ^a–d^	0.50 ± 0.05 ^b–d^	17.88 ± 1.92 ^a–c^	4.71 ± 0.47 ^a^	29.6 ± 2.9 ^ab^
Regina/M × M 14		0.04 ± 0.005 ^b–d^	0.12 ± 0.01 ^a–c^	0.09 ± 0.02 ^c^	0.42 ± 0.05 ^a–d^	0.49 ± 0.07 ^b–d^	14.80 ± 1.64 ^a–d^	4.37 ± 0.41 ^a^	25.7 ± 1.5 ^a–c^
Regina/Gisela 6		0.04 ± 0.005 ^b–d^	0.17 ± 0.04 ^a–c^	0.19 ± 0.02 ^a–c^	0.27 ± 0.02 ^d^	0.33 ± 0.03 ^cd^	10.23 ± 1.46 ^cd^	4.61 ± 0.28 ^a^	19.5 ± 2.3 ^cd^
Cultivar	Carmen	0.03 ± 0.002 ^b^	0.10 ± 0.01	0.19 ± 0.03 ^a^	0.48 ± 005	0.40 ± 0.04 ^b^	11.76 ± 0.81 ^b^	1.16 ± 0.06 ^b^	14.7 ± 1.2 ^c^
	Kordia	0.07 ± 0.006 ^a^	0.14 ± 0.01	0.17 ± 0.02 ^b^	0.50 ± 0.06	0.72 ± 0.07 ^a^	17.37 ± 1.20 ^a^	2.98 ± 0.10 ^ab^	22.8 ± 1.7 ^a^
	Regina	0.04 ± 0.002	0.04 ± 0.01	0.12 ± 0.01 ^c^	0.38 ± 0.03	0.38 ± 0.03 ^b^	12.33 ± 1.03 ^b^	3.94 ± 0.24 ^a^	18.2 ± 1.4 ^b^
Rootstock	Mahaleb	0.04 ± 0.007	0.08 ± 0.01 ^c^	0.08 ± 0.02 ^c^	0.37 ± 0.04 ^b^	0.30 ± 0.04 ^b^	8.79 ± 1.01 ^c^	2.24 ± 0.23 ^d^	16.2 ± 2.6 ^b^
	Colt	0.05 ± 0.002	0.13 ± 0.02 ^a^	0.17 ± 0.03 ^b^	0.42 ± 0.04 ^b^	0.60 ± 0.11 ^a^	16.37 ± 1.63 ^a^	2.35 ± 0.29 ^c^	27.0 ± 3.1 ^a^
	Oblacinska	0.05 ± 0.004	0.15 ± 0.03 ^a^	0.16 ± 0.03 ^b^	0.46 ± 0.05 ^ab^	0.54 ± 0.09 ^a^	15.13 ± 1.51 ^ab^	2.97 ± 0.40 ^a^	26.2 ± 2.8 ^a^
	M × M 14	0.05 ± 0.011	0.09 ± 0.01 ^bc^	0.10 ± 0.01 ^bc^	0.39 ± 0.03 ^b^	0.53 ± 0.06 ^a^	15.42 ± 1.15 ^ab^	2.76 ± 0.37 ^ab^	25.9 ± 2.0 ^a^
	Gisela 5	0.05 ± 0.006	0.15 ± 0.02 ^a^	0.28 ± 0.04 ^a^	0.64 ± 0.13 ^a^	0.59 ± 0.09 ^a^	14.89 ± 1.27 ^ab^	1.91 ± 0.25 ^d^	24.6 ± 2.9 ^a^
	Gisela 6	0.05 ± 0.008	0.14 ± 0.02 ^a^	0.18 ± 0.04 ^b^	0.47 ± 0.10 ^ab^	0.45 ± 0.06 ^ab^	12.33 ± 1.73 ^bc^	3.86 ± 0.28 ^a^	24.2 ± 2.5 ^ab^
Year	2020	0.05 ± 0.001	0.14 ± 0.01 ^a^	0.18 ± 0.02 ^a^	0.51 ± 0.05	0.62 ± 0.05 ^a^	15.60 ± 1.00 ^a^	2.45 ± 0.16 ^b^	20.1 ± 1.3 ^a^
	2021	0.04 ± 0.002	0.11 ± 0.01 ^b^	0.14 ± 0.01 ^b^	0.42 ± 0.04	0.39 ± 0.03 ^b^	12.21 ± 0.75 ^b^	2.79 ± 0.22 ^a^	16.6 ± 1.0 ^b^
Statistical significance									
Cultivar		***	***	ns	ns	***	***	***	***
Rootstock		ns	ns	***	*	***	***	***	***
Year		ns	ns	*	ns	***	***	***	***
Cultivar × Rootstock		*	*	*	***	*	*	***	*

Data are presented as means ± standard errors (*n* = 3). Different superscript letter in a same column (factor) denotes significant difference (Tukey’s test, *p* < 0.05). Statistical significance: ns—not significant; * *p* < 0.05; *** *p* < 0.001.

**Table 5:** Content of individual and total flavanones in fruits of sweet cherry cultivars grafted on different rootstocks (mg/kg FW, average 2020–2021).

Combination Cultivar/Rootstock		Naringenin Hexoside 1	Naringenin Hexoside 2	Taxifolin Hexoside	Taxifolin Rutinoside	Total Flavanones
Carmen/Mahaleb		0.08 ± 0.01	0.13 ± 0.04 ^bc^	1.89 ± 0.41 ^ef^	0.99 ± 0.10 ^c–e^	3.09 ± 0.36 ^de^
Carmen/Colt		0.12 ± 0.02	0.11 ± 0.02 ^c^	1.99 ± 0.56 ^d–f^	1.27 ± 0.16 ^ab^	3.49 ± 0.21 ^b–e^
Carmen/Oblacinska		0.13 ± 0.02	0.19 ± 0.03 ^a–c^	2.53 ± 0.56 ^b–f^	1.38 ± 0.09 ^a^	4.23 ± 0.44 ^ab^
Carmen/M× M 14		0.06 ± 0.02	0.11 ± 0.02 ^c^	2.80 ± 0.15 ^a–e^	0.68 ± 0.04 ^fg^	3.65 ± 0.30 ^b–d^
Carmen/Gisela 5		0.09 ± 0.03	0.17 ± 0.03 ^a–c^	3.47 ± 0.29 ^ab^	0.85 ± 0.03 ^d–q^	4.58 ± 0.33 ^ab^
Carmen/Gisela 6		0.09 ± 0.03	0.24 ± 0.06 ^a–c^	3.34 ± 0.11 ^ab^	0.98 ± 0.04 ^c–d^	4.65 ± 0.37 ^a^
Kordia/Mahaleb		0.07 ± 0.02	0.10 ± 0.04 ^c^	1.61 ± 0.25 ^f^	0.77 ± 0.04 ^d–g^	2.55 ± 0.34 ^e^
Kordia’/Colt		0.05 ± 0.01	0.10 ± 0.02 ^c^	3.61 ± 0.41 ^a^	0.72 ± 0.08 ^c–g^	4.51 ± 0.26 ^ab^
Kordia/Oblacinska		0.11 ± 0.01	0.23 ± 0.02 ^a–c^	2.38 ± 0.48 ^c–f^	1.24 ± 0.13 ^a–c^	3.96 ± 0.33 ^b–d^
Kordia/M × M 14		0.07 ± 0.01	0.17 ± 0.03 ^a–c^	2.33 ± 0.25 ^c–f^	1.18 ± 0.07 ^a–c^	3.75 ± 0.18 ^b–d^
Kordia/Gisela 5		0.08 ± 0.01	0.06 ± 0.01 ^c^	2.72 ± 0.38 ^a–e^	1.18 ± 0.12 ^a–c^	4.04 ± 0.22 ^a–c^
Kordia/Gisela 6		0.07 ± 0.02	0.33 ± 0.04 ^ab^	3.22 ± 0.37 ^a–c^	1.02 ± 0.09 ^b–d^	4.16 ± 0.17 ^a–d^
Regina/Mahaleb		0.05 ± 0.02	0.24 ± 0.05 ^a–c^	3.60 ± 0.47 ^a^	0.95 ± 0.06 ^d–f^	4.64 ± 0.36 ^a^
Regina/Colt		0.06 ± 0.01	0.13 ± 0.03 ^bc^	3.40 ± 0.45 ^ab^	0.74 ± 0.04 ^e–g^	4.33 ± 0.13 ^a–c^
Regina/Oblacinska		0.07 ± 0.01	0.13 ± 0.03 ^bc^	2.72 ± 0.28 ^a–e^	0.68 ± 0.03 ^fg^	3.64 ± 0.23 ^b–d^
Regina/M × M 14		0.11 ± 0.01	0.15 ± 0.03 ^a–c^	2.92 ± 0.14 ^a–d^	0.78 ± 0.06 ^d–g^	4.56 ± 0.36 ^b–d^
Regina/Gisela 6		0.14 ± 0.03	0.35 ± 0.02 ^a^	3.22 ± 0.14 ^a–c^	0.65 ± 0.02 ^g^	4.36 ± 0.17 ^a–d^
Cultivar	Carmen	0.11 ± 0.01 ^a^	0.11 ± 0.01 ^b^	2.31 ± 0.19 ^b^	1.21 ± 0.07 ^a^	3.78 ± 0.16 ^b^
	Kordia	0.07 ± 0.01 ^b^	0.24 ± 0.03 ^a^	3.31 ± 0.12 ^a^	0.87 ± 0.04 ^ab^	4.58 ± 0.18 ^a^
	Regina	0.09 ± 0.02 ^ab^	0.18 ± 0.03 ^a^	2.82 ± 0.18 ^ab^	0.72 ± 0.02 ^b^	3.85 ± 0.20 ^ab^
Rootstock	Mahaleb	0.07 ± 0.01	0.14 ± 0.02 ^b^	2.10 ± 0.19 ^b^	0.81 ± 0.05 ^c^	3.20 ± 0.19 ^b^
	Colt	0.06 ± 0.01	0.24 ± 0.05 ^a^	2.88 ± 0.25 ^a^	0.94 ± 0.06 ^b^	4.23 ± 0.29 ^a^
	Oblacinska	0.09 ± 0.01	0.22 ± 0.05 ^a^	3.03 ± 0.29 ^a^	0.95 ± 0.08 ^b^	4.39 ± 0.26 ^a^
	M × M 14	0.08 ±0.01	0.12 ± 0.02 ^b^	3.01 ± 0.19 ^a^	0.90 ± 0.06 ^bc^	4.21 ± 0.20 ^a^
	Gisela 5	0.11 ± 0.02	0.18 ± 0.04 ^ab^	2.93 ± 0.29 ^a^	1.18 ± 0.15 ^a^	4.60 ± 0.26 ^a^
	Gisela 6	0.11 ± 0.03	0.13 ± 0.02 ^b^	2.94 ± 0.26 ^a^	0.97 ± 0.11 ^b^	4.26 ± 0.25 ^a^
Year	2020	0.09 ± 0.01	0.17 ± 0.02	1.05 ± 0.06 ^a^	0.72 ± 0.02 ^a^	2.39 ± 0.17 ^b^
	2021	0.08 ± 0.01	0.17 ± 0.02	0.84 ± 0.03 ^b^	0.17 ± 0.02 ^b^	3.23 ± 0.09 ^a^
Statistical significance						
Cultivar		*	***	***	***	***
Rootstock		ns	***	***	***	***
Year		ns	ns	***	***	***
Cultivar × Rootstock		ns	ns	***	***	*

Data are presented as means ± standard errors (*n* = 3). Different superscript letter in a same column (factor) denotes significant difference (Tukey’s test, *p* < 0.05). Statistical significance: ns—not significant; * *p* < 0.05; *** *p* < 0.001.

**Table 6:** Content of individual and total hydroxycinnamic acids in fruits of sweet cherry cultivars on different rootstocks (mg/kg of FW, average 2020–2021).

Comination Cultivar/Rootstock		Caffeic Acid Derivatives	Caffeoyl- Quinic Acid Derivatives	Coumaroyl- Quinic Acid Derivatives	Dicaffeoyl- Quinic Acids	Ferulic Acid Derivatives	Feruloyl- Quinic Acid Derivatives	Sinapic Acid Derivatives	*p*-Coumaric Acid Derivatives	Total Hydroxycin- Namic Acids
Carmen/Mahaleb		29.6 ± 2.9 ^d–f^	43.4 ± 5.0	31.0 ± 3.7 ^d–g^	0.030 ± 0.002	0.32 ± 0.03 ^de^	1.05 ± 0.06	0.21 ± 0.02 ^b–d^	5.48 ± 0.92 ^e–h^	111.1 ± 14.8 ^fg^
Carmen/Colt		52.0 ± 5.5 ^b–f^	59.4 ± 5.3	33.7 ± 2.6 ^d–f^	0.027 ± 0.003	0.39 ± 0.04 ^c–e^	1.27 ± 0.05	0.25 ± 0.02 ^ab^	9.66 ± 1.02 ^c–e^	156.7 ± 19.5 ^b–d^
Carmen/Oblacinska		36.7 ± 4.2 ^d–g^	63.2 ± 7.5	58.6 ± 3.6 ^a^	0.019 ± 0.003	0.34 ± 0.04 ^de^	1.29 ± 0.07	0.27 ± 0.03 ^ab^	6.81 ± 1.71 ^d–h^	167.2 ± 21.7 ^ab^
Carmen/M × M 14		50.0 ± 5.1 ^b–f^	61.1 ± 7.0	38.6 ± 3.1 ^c–e^	0.021 ± 0.003	0.46 ± 0.06 ^a–e^	1.32 ± 0.06	0.25 ± 0.03 ^a–c^	9.29 ± 1.29 ^c–f^	161,1 ± 19.8 ^a–c^
Carmen/Gisela 5		42.4 ± 4.7 ^c–g^	87.0 ± 7.5	52.4 ± 4.9 ^ab^	0.026 ± 0.003	0.42 ± 0.04 ^a–e^	1.45 ± 0.14	0.29 ± 0.03 ^a^	7.87 ± 0.81 ^d–g^	190.9 ± 26.8 ^a^
Carmen/Gisela 6		29.0 ± 4.6 ^e–g^	46.3 ± 4.4	40.9 ± 3.9 ^b–d^	0.028 ± 0.002	0.40 ± 0.05 ^b–e^	1.32 ± 0.07	0.26 ± 0.02 ^ab^	5.36 ± 0.85 ^f–h^	123.6 ± 14.1 ^de^
Kordia/Mahaleb		46.0 ± 3.6 ^c–g^	55.9 ± 1.4	27.6 ± 1.4 ^e–g^	0.029 ± 0.004	0.47 ± 0.05 ^a–e^	0.97 ± 0.03	0.14 ± 0.01 ^g^	8.54 ± 0.68 ^c–f^	139.6 ± 15.0 ^c–e^
Kordia/Colt		86.1 ± 9.6 ^a^	61.8 ± 8.3	32.3 ± 3.4 ^d–g^	0.033 ± 0.002	0.60 ± 0.06 ^ab^	1.34 ± 0.13	0.20 ± 0.01 ^c–f^	16.02 ± 1.03 ^a^	198.4 ± 17.8 ^a^
Kordia/Oblacinska		57.6 ± 5.5 ^a–e^	69.4 ± 4.8	39.4 ± 3.3 ^b–e^	0.036 ± 0.006	0.58 ± 0.05 ^a–c^	1.23 ± 0.07	0.18 ± 0.01 ^d–g^	10.70 ± 1.96 ^b–d^	179.1 ± 19.9 ^ab^
Kordia/M × M 14		77.0 ± 7.8 ^ab^	64.4 ± 6.3	29.9 ± 2.6 ^d–g^	0.031 ± 0.009	0.57 ± 0.05 ^a–c^	1.12 ± 0.07	0.16 ± 0.01 ^d–g^	14.32 ± 1.83 ^ab^	187.5 ±2 5.8 ^ab^
Kordia/Gisela 5		58.0 ± 6.9 ^a–d^	72.2 ± 8.7	48.0 ± 4.4 ^a–c^	0.036 ± 0.009	0.56 ± 0.02 ^a–c^	1.31 ± 0.02	0.21 ± 0.01 ^c–e^	10.77 ± 1.41 ^b–d^	191.1 ± 22.4 ^a^
Kordia/Gisela 6		37.5 ± 5.7 ^d^–^g^	60.1 ± 5.1	48.3 ± 5.0 ^a–c^	0.026 ± 0.007	0.54 ± 0.06 ^a–c^	1.31 ± 0.20	0.21 ± 0.03 ^c–f^	6.94 ± 1.08 ^d–h^	154.4 ± 18.7 ^b–d^
Regina/Mahaleb		18.4 ± 1.8 ^g^	42.7 ± 3.3	10.2 ± 1.4 ^h^	0.034 ± 0.004	0.27 ± 0.04 ^e^	0.81 ± 0.07	0.16 ± 0.01 ^e–g^	3.40 ± 0.33 ^h^	75.9 ± 13.2 ^g^
Regina/Colt		43.7 ± 4.2 ^c–g^	46.1 ± 4.5	23.3 ± 3.1 ^f–h^	0.035 ± 0.005	0.46 ± 0.05 ^a–e^	0.96 ± 0.08	0.14 ± 0.01 ^g^	8.11 ± 1.72 ^d–g^	122.8 ± 15.9 ^ef^
Regina/Oblacinska		66.9 ± 6.6 ^a–c^	47.6 ± 5.7	27.9 ± 5.2 ^d–g^	0.038 ± 0.004	0.61 ± 0.07 ^a^	1.14 ± 0.03	0.15 ± 0.02 ^fg^	12.43 ± 1.18 ^a–c^	156.8 ± 26.1 ^b–d^
Regina/M × M 14		68.0 ± 5.8 ^a–c^	49.4 ± 4.7	22.1 ± 3.7 ^f–h^	0.041 ± 0.008	0.49 ± 0.05 ^a–d^	1.06 ± 0.08	0.16 ± 0.01 ^e–g^	12.66 ± 1.70 ^ab^	153.9 ± 23.2 ^b–e^
Regina/Gisela 6		23.4 ± 2.9 ^fg^	42.6 ± 4.8	19.6 ± 1.8 ^gh^	0.038 ± 0.008	0.57 ± 0.05 ^a–c^	1.01 ± 0.09	0.16 ± 0.03 ^d–g^	4.00 ± 0.50 ^gh^	91.4 ± 24.4 ^fg^
Cultivar	Carmen	55.8 ± 6.1 ^a^	62.0 ± 4.2 ^a^	36.3 ± 2.4 ^a^	0.034 ± 0.002 ^a^	0.39 ± 0.03 ^c^	1.27 ± 0.05 ^a^	0.25 ± 0.01	7.41 ± 0.56 ^b^	163.4 ± 9.9 ^a^
	Kordia	52.9 ± 4.6 ^a^	50.8 ± 2.9 ^b^	32.7 ± 2.4 ^c^	0.028 ± 0.002 ^b^	0.56 ± 0.02 ^a^	1.09 ± 0.04 ^c^	0.18 ± 0.01	11.21 ± 1.01 ^a^	154.5 ± 15.0 ^a^
	Regina	34.2 ± 2.4 ^b^	60.9 ± 2.3 ^a^	33.9 ± 3.6 ^b^	0.032 ± 0.003 ^ab^	0.48 ± 0.03 ^b^	1.16 ± 0.05 ^bc^	0.16 ± 0.01	8.12 ± 1.14 ^b^	139.9 ± 13.5 ^b^
Rootstock	Mahaleb	31.3 ± 3.4 ^c^	44.0 ± 3.4 ^c^	22.9 ± 2.6 ^e^	0.031 ± 0.002	0.35 ± 0.03 ^b^	0.95 ± 0.04 ^c^	0.17 ± 0.01 ^c^	5.81 ± 0.63 ^c^	105.5 ± 17.4 ^c^
	Colt	60.6 ± 6.8 ^ab^	62.4 ± 6.5 ^ab^	29.8 ± 2.0 ^de^	0.032 ± 0.003	0.48 ± 0.04 ^a^	1.19 ± 0.06 ^b^	0.20 ± 0.01 ^b^	11.26 ± 1.63 ^ab^	155.9 ± 24.2 ^b^
	Oblacinska	53.7 ± 6.5 ^ab^	60.1 ± 4.2 ^ab^	41.9 ± 3.8 ^b^	0.031 ± 0.004	0.51 ± 0.05 ^a^	1.22 ± 0.03 ^b^	0.20 ± 0.02 ^b^	9.98 ± 1.21 ^ab^	163.64 ± 17.2 ^ab^
	M × M 14	65.0 ± 5.6 ^a^	63.3 ± 4.5 ^a^	30.2 ± 2.4 ^cd^	0.031 ± 0.004	0.51 ± 0.03 ^a^	1.17 ± 0.05 ^b^	0.19 ± 0.01 ^bc^	12.09 ± 1.42 ^a^	172.49 ± 21.8 ^b^
	Gisela 5	50.2 ± 6.0 ^b^	69.1 ± 5.5 ^a^	50.2 ± 4.4 ^a^	0.031 ± 0.004	0.49 ± 0.05 ^a^	1.38 ± 0.07 ^a^	0.25 ± 0.03 ^a^	9.32 ± 1.50 ^b^	180.97 ± 22.0 ^a^
	Gisela 6	30.0 ± 2.8 ^c^	51.3 ± 3.0 ^bc^	36.3 ± 4.7 ^bc^	0.03 1± 0.003	0.50 ± 0.05 ^a^	1.21 ± 0.08 ^b^	0.21 ± 0.02 ^b^	5.43 ± 0.54 ^c^	124.98 ± 21.5 ^c^
Year	2020	56.8 ± 5.8 ^a^	54.0 ± 3.7 ^b^	26.3 ± 2.9 ^b^	0.033 ± 0.002	0.41 ± 0.03 ^b^	1.18 ± 0.29	0.22 ± 0.01 ^a^	10.53 ± 0.99 ^a^	149.5 ± 11.1 ^b^
	2021	40.0 ± 2.5 ^b^	61.5 ± 1.8 ^a^	42.3 ± 2.7 ^a^	0.029 ± 0.002	0.54 ± 0.02 ^a^	1.17 ± 0.22	0.18 ± 0.01 ^b^	7.40 ± 0.37 ^b^	153.1 ± 10.1 ^a^
Statistical significance										
Cultivar		***	***	***	***	***	***	***	***	***
Rootstock		***	***	***	ns	***	***	**	***	***
Year		***	***	***	ns	***	ns	***	***	***
Cultivar × Rootstock		***	ns	***	ns	**	ns	*	***	*

Data are presented as means ± standard errors (*n* = 3). Different superscript letter in a same column (factor) denotes significant difference (Tukey’s test, *p* < 0.05). Statistical significance: ns—not significant; * *p* < 0.05; ** *p* < 0.01; *** *p* < 0.001.

## Supplementary Materials

The following supporting information can be downloaded at: https://www.mdpi.com/article/10.3390/plants12010103/s1, Table S1: All 54 identified phenolic compounds in sweet cherry fruits and their abbreviations; Table S2: Content of all individual and total flavanols in fruits of sweet cherry cultivars on different rootstocks; Table S3: Content of all individual and total hydroxycinnamic acids in fruits of sweet cherry cultivars on different rootstocks; Table S4: Meteorological conditions during the first six months in the years of study (2020–2021).
