# Supplementary material for: The Phenolic Profile of Sweet Cherry Fruits Influenced by Cultivar/Rootstock Combination
Source: Plants (Basel). 2022 Dec 25;12(1):103. doi: 10.3390/plants12010103 (PMC9823671; doi:10.3390/plants12010103)
Supplement: Supplementary file 1 [file plants-12-00103-s001.zip › plants-2091149-supplementary.pdf]

Table S1. Identification of phenolic compounds in cherry fruits in positive and negative ionization with HPLC-MS and MS^2/MS^3

Phenolic compounds	Abbreviation	λmax (nm)	[M-H] *[M+H]+ (*m/z*)*	MS^2 (m/z)	MS^3(m/z)
**Anthocyanins**					
Cyanidin-3-rutinoside	cy-3-rut	280, 522	595	449/287	
Cyanidin-3-glucoside	cy-3-glu	280, 519	449	287	
Pelargonidin-3-rutinoside	pe-3-rut	278, 504	579	433/271	
Peonidin-3-rutinoside	peo-3-rut	280, 523	609	463/301	
**Flavanols**					
Quercetin-7-glucoside-3-rutinoside	qu-7-glu-3-rut	256, 355	771	609	301
Quercetin-3-rutinoside	qu-3-rut	255,355	609	301	
Quercetin-3-galactoside	qu-3-gal	256,356	463	301	
Quercetin-3-glucoside	qu-3-glu	255,355	463	301	
Kaempferol-3-rutinoside	kae-3-rut	264,345	593	285	
Isorhamnetin-3-rutinoside	iso-3-rut	256, 354	623	315	
Kaempferol-3-glucoside	kae-3-glu	266,298,346	447	285	
**Flavanols**					
Procyanidin dimer 1	pro dim 1	235,278	577	425,451,407,289	
Procyanidin dimer 2	pro dim 2	234,278	577	425,451,407,289	
Catechin	cat	234, 279	289	245	
Procyanidin trimer 1	pro tri 1	234,278	865	577,407,289	
Procyanidin dimer 3	pro dim 3	235,279	577	425,407,289	
Procyanidin dimer 4	pro dim 4	235, 280	577	425,451,407,289	
Epicatechin	epicat	234,279	289	245	
Procyanidin trimer 2	pro tri 2	234, 280	865	739, 695, 577,451, 425,407, 289	
Procyanidin dimer 5	pro dim 5	234,279	577	451, 425,407,289	
Procyanidin dimer 6	pro dim 6	234,279	577	425,407,289	
Epicatechin gallate	epicat gal	234, 280	441	289	
**Flavanones**					
Naringenin hexoside 1	na hex 1	213, 225, 289	433	271	
Naringenin hexoside 2	na hex 2	213, 225, 289	433	271	
Taxifolin rutinoside	tax rut	286	611	285, 485, 475, 241,303	
Taxifolin hexoside	tax hex	288	465	285, 241, 417, 303	
**Hydroxycinnamic acids**					
3-*p*-coumaroylquinic acid	3-p-CoQA	312	337	163	
4-*p*-coumaroylquinic acid	4-CoQA	312	337	173, 163, 155, 137, 191	
Dicaffeoylquinic acid 1	di CQA 1	246, 316	515	353	191
Dicaffeoylquinic acid 2	di CQA 2	247, 321	515	353	191, 179
Caffeoylquinic acid glycoside 1	CQA gly 1	290, 325	515	341, 353, 335, 179, 191	
Caffeoylquinic acid glycoside 2	CQA gly 2	290, 324	515	341, 353, 173, 179	
Caffeic acid glycoside 1	CA gly 1	293, 325	341	179,135	
cis-3-caffeoylquinic acid (cis3CQA)	c-3- CQA	234, 326	353	191, 179, 135	173, 127, 85
Neochlorogenic acid (*trans*3CQA)	neoCQA	234, 326	353	191,179,135	173, 127, 85
Caffeic acid hexoside 1	CA hex 1	330	341	179	
Caffeoylquinic acid glycoside 3	CQA gly 3	290, 326	515	341, 353, 179, 173	
Caffeic acid hexoside 2	CA hex 2	330	341	179	
p-coumaric acid hexoside	p-couQA hex	311	325	163	
trans 3-p-coumaroylquinic acid	t-3-p-CoQA,	312	337	163	
trans 4-caffeoylquinic acid	t-4-p-CQA	234,328	353	173,179,191,135	
trans 5-caffeoylquinic acid	t-5-CQA	234,328	353	191,179,173,135	
cis-3-p-coumaroylquinic acid	c-3-p-CoQA	312	337	163	
cis 3-feruloyquinic acid	c-3-FQA	320	367	193,134	
cis 4-caffeoylquinic acid	c-4-CQA	234,328	353	173,179,191,135	149
trans 3-feruloyquinic acid	t-3-FQA	320	367	193,134	149
Sinapic acid hexoside	sin A hex	236, 326	385	223, 205, 191	
trans-5-feruloylquinic acid	t-5-FQA	322	367	193, 134	
Caffeic acid glycoside 2	CA gly 2	293, 326	341	179,135	
Feruloyl hexoside	Fhex	328	355	193,217,295,175,235	
cis 5-caffeoylquinic acid	c-5-CQA	234,328	353	191,179,135	
trans 4-*p*-coumaroylquinic acid	t-4-p-CoQA	312	337	173, 163, 155, 137, 191	
cis-4-*p*-coumaroylquinic acid	c-4-p-CoQA	311	337	173, 163, 137, 191	
5-*p*-coumaroylquinic acid	5-p-CpQA	311	337	191,163,173	

*[M+H]+ (*m/z*) anthocyanins were obtained in the positive ion mode, other phenolics in the negative ion mode

Table S2. Content of all individual and total flavanols in fruits of sweet cherry cultivars on different rootstocks (mg/kg of fresh weight, average 2020-2021).

Combination cultivar/rootstock		Procyanidin dimer 6	Procyanidin dimer 1	Procyanidin dimer 2	Catechin	Procyanidin trimer 1	Procyanidin dimer 3	Procyanidin dimer 4	Epicatechin	Procyanidin trimer 2	Procyanidin dimer 5	Epicatechin gallate	Total flavanols
Carmen/Mahaleb		0.33±0.02^{b-d}	3.96±0.73^{ef}	1.11±0.18^{d-f}	2.13±0.28^{c-f}	1.9±0.25^{c-f}	0.35±0.08^{fg}	3.77±0.39^{fg}	7.87±0.84^{b-d}	6.17±0.75^{i}	1.06±0.31^{f}	0.75±0.21^{f}	29.4±6.3^{e}
Carmen/Colt		0.50±0.08^{a-d}	4.61±0.34^{c-f}	1.94±0.21^{b-e}	2.67±0.12^{a-e}	2.38±0.11^{a-e}	0.44±0.04^{d-g}	4.46±0.50^{d-g}	10.65±0.94^{a-c}	4.76±0.23^{i}	1.15±0.13^{f}	0.90±0.23^{f}	34.4±6.5^{de}
Carmen/Oblacinska		0.42±0.04^{a-d}	5.74±0.33^{a-e}	1.37±0.34^{d-f}	3.14±0.16^{a-c}	2.80±0.14^{a-c}	0.37±0.10^{e-g}	3.35±0.41^{g}	11.83±0.71^{ab}	9.74±0.61^{f-h}	1.65±0.27^{d-f}	2.28±0.33^{b-d}	40.2±3.9^{b-e}
Carmen/M×M 14		0.38±0.07^{b-d}	5.39±0.26^{b-f}	1.87±0.19^{b-e}	2.49±0.12^{a-f}	2.22±0.10^{a-f}	0.51±0.07^{a-f}	4.23±0.41^{d-g}	11.34±0.72$_{ab}$	6.05±0.49^{i}	1.32±0.17^{ef}	0.67±0.14^{f}	36.51±7.9^{c-e}
Carmen/Gisela 5		0.44±0.05 $^{a-d}$	6.48±0.68^{a-d}	1.58±0.36^{c-f}	3.04±0.29^{a-d}	2.71±0.26^{a-d}	0.47±0.11^{b-g}	3.83±0.48^{e-g}	10.19±1.24^{a-c}	7.15±0.85^{hi}	1.87±0.29^{c-f}	1.30±0.28^{d-f}	39.1±3.4^{b-e}
Carmen/Gisela 6		0.78±0.24^{a}	3.96±0.42^{ef}	1.08±0.17^{d-f}	2.58±0.24^{a-f}	2.30±0.21^{a-f}	0.45±0.03^{c-g}	3.44±0.46^{fg}	8.90±0.48^{a-d}	7.32±1.15^{hi}	1.49±0.33^{d-f}	1.15±0.20^{ef}	33.4±7.4^{de}
Kordia/Mahaleb		0.41±0.12^{a-d}	5.16±0.56^{b-f}	1.72±0.14^{c-f}	1.93±0.07^{d-f}	1.73±0.06^{d-f}	0.52±0.03^{a-f}	4.61±0.16^{c-g}	8.10±1.04^{b-d}	12.82±0.86^{b-f}	2.44±0.21^{c-e}	1.68±0.31^{c-f}	41.1±4.3^{b-d}
Kordia/Colt		0.44±0.06^{a-d}	7.90±0.95^{a}	3.22±0.41^{a}	3.63±0.27^{a}	3.24±0.28^{a}	0.67±0.09^{a}	7.07±0.51^{ab}	14.04±1.69^{a}	14.64±1.63^{b-d}	2.57±0.42^{c-e}	1.63±0.28^{c-f}	59.1±4.9^{a}
Kordia/Oblacinska		0.41±0.08^{a-d}	7.08±0.53^{a-c}	2.15±0.39^{a-d}	2.99±0.29^{a-d}	2.67±0.26^{a-d}	0.65±0.05^{ab}	4.92±0.45^{c-g}	9.13±1.16^{a-d}	15.27±1.32^{bc}	2.94±0.20^{bc}	2.33±0.20^{b-d}	50.4±5.9^{ab}
Kordia/M×M 14		0.38±0.07^{b-d}	7.41±0.50^{ab}	2.88±0.37^{ab}	2.31±0.38^{b-f}	2.06±0.34^{b-f}	0.64±0.05^{ab}	5.95±0.44^{a-d}	8.85±0.79^{a-d}	11.26±0.93^{e-g}	2.05±0.32^{c-f}	1.44±0.21^{c-f}	45.2±6.1^{bc}
Kordia/Gisela 5		0.70±0.19^{ab}	6.19±0.63^{a-d}	2.17±0.28^{a-d}	3.43±0.17^{ab}	3.06±0.15ab	0.63±0.02^{a-c}	5.74±0.38^{a-e}	11.88±1.84^{ab}	19.08± 0.58^{a}	3.93±0.34^{b}	2.45±0.19^{a-c}	59.2±9.1^{a}
Kordia/Gisela 6		0.66±0.17^{a-c}	4.78±0.49^{c-f}	1.40±0.22^{d-f}	3.18±0.33^{a-c}	2.84±0.26^{a-c}	0.60±0.07^{a-d}	5.19±0.37^{b-f}	10.57±1.32^{a-c}	15.51±0.28^{b}	3.13±0.21^{bc}	2.02±0.18^{b-e}	49.9±5.8^{ab}
Regina/Mahaleb		0.36±0.05^{b-d}	3.16±0.32^{f}	0.68±0.07^{f}	0.71±0.07^{g}	0.64±0.06^{g}	0.30±0.05^{g}	5.34±0.43^{b-f}	4.55±0.98^{d}	9.47±0,65^{gh}	2.51±0.31^{c-e}	1.97±0.31^{b-e}	29.7±3.5^{e}
Regina/Colt		0.32±0.03^{cd}	4.41±0.48^{d-f}	1.63±0.35^{c-f}	1.70±0.35^{e-g}	1.52±0.31^{e-g}	0.51±0.08^{a-f}	6.91±0.45^{ab}	9.55±1.57^{a-d}	11.67±1.38^{d-g}	2.59±0.32^{c-e}	2.04±0.29^{b-e}	42.9±5.9^{b-d}
Regina/Oblacinska		0.54±0.11^{a-d}	6.06±0.81^{a-d}	2.50±0.44^{a-c}	2.29±0.33^{b-f}	2.04±0.29^{b-f}	0.68±0.08^{a}	6.54±0.43^{a-c}	11.73±1.08^{ab}	12.06±1.11^{c-g}	3.10±0.27^{bc}	3.00±0.27^{ab}	50.5±5.4^{ab}
Regina/M×M 14		0.42±0.04^{a-d}	4.14±0.44^{d-f}	2.55±0.35^{a-c}	1.60±0.38^{e-g}	1.43±0.33^{e-g}	0.55±0.05^{ab}	7.53±0.31^{a}	7.30±1.06^{b-d}	11.25±0.87^{e-g}	2.75±0.23^{b-d}	2.47±0.25^{a-c}	41.9±6.7^{b-d}
Regina/Gisela 6		0.25± 0.01^{d}	5.38±0.43^{b-f}	0.86±0.13^{ef}	1.44±0.13^{fg}	1.32±0.13^{fg}	0.59±0.03^{a-d}	4.63±0.19^{c-g}	5.46±0.61^{cd}	13.04±0.71^{b-e}	5.21±0.28^{a}	3.48±0.13^{a}	41.6±4.5^{b-d}
Cultivar	Carmen	0.47±0.05^{ab}	5.02±0.35^{b}	1.49±0.11^{b}	2.67±0.11^{a}	2.38±0.09^{a}	0.43±0.02^{c}	3.84±0.18^{c}	10.13±0.62^{ab}	6.86±0.45^{c}	1.42 ± 0.11^{c}	1.17±0.13^{c}	37.29±1.69^{c}
	Kordia	0.50±0.06^{a}	6.42±0.42^{a}	2.26±0.21^{a}	2.97±0.23^{a}	2.60±0.02^{a}	0.62±0.02^{a}	5.58±0.24^{b}	10.43±0.73^{a}	14.76±0.69^{a}	2.84 ± 0.15^{b}	1.93±0.11^{b}	52.84±3.49^{a}
	Regina	0.38±0.03^{b}	4.63±0.33^{b}	1.65±0.23^{ab}	1.55±0.15^{b}	1.39±0.13^{b}	0.53±0.03^{b}	6.19±0.37^{a}	7.72±0.75^{b}	11.50±0.87^{b}	3.23 ± 0.29^{a}	2.59±0.17^{a}	43.33±2.62^{b}
Rootstock	Mahaleb	0.37±0.04^{b}	4.09±0.46^{b}	1.17±0.13^{de}	1.59±0.18^{c}	1.42±0.16^{c}	0.39±0.04^{d}	4.57±0.28^{b}	6.84±0.85^{b}	9.49±0.82^{b}	2.00±0.25^{b}	1.47±0.21^{b}	36.12±2.67^{c}
	Colt	0.42±0.04^{b}	5.64±0.59^{a}	2.27±0.33^{ab}	2.67±0.33^{a}	2.38±0.29^{ab}	0.54±0.05^{bc}	6.14±0.46^{a}	11.41±1.64^{a}	10.36±0.51^{b}	2.10±0.31^{b}	1.52±0.22^{b}	49.50±5.81^{ab}
	Oblacinska	0.46±0.05^{b}	6.29±0.53^{a}	2.01±0.24^{bc}	2.81±0.17^{a}	2.50±0.05^{a}	0.57±0.05^{b}	4.94±0.43^{b}	10.90±0.97^{a}	12.36±0.79^{a}	2.56±0.26^{b}	2.54±0.17^{a}	51.66±3.88^{a}
	MxM 14	0.39±0.03^{b}	5.64±0.58^{a}	2.43±0.29^{a}	2.13±0.19^{bc}	1.90±0.17^{bc}	0.57±0.03^{b}	5.90±0.56^{a}	9.17±0.58^{ab}	9.52±0.91^{b}	2.04±0.25^{b}	1.52±0.21^{b}	44.67±4.33^{bc}
	Gisela 5	0.67±0.12^{a}	5.26±0.57^{ab}	1.67±0.23^{cd}	2.82±0.18^{a}	2.52±0.17^{a}	0.47±0.05^{c}	4.57±0.32^{b}	10.89±1.21^{a}	12.12±0.55^{a}	2.56±0.32^{b}	1.77±0.19^{b}	49.10±6.01^{ab}
	Gisela 6	0.42±0.10^{b}	5.51±0.34^{a}	1.05±0.10^{e}	2.60±0.20^{ab}	2.34±0.25^{ab}	0.66±0.03^{a}	4.56±0.32^{b}	7.15±0.65^{b}	12.87±0.71a	3.98±0.41^{a}	2.54±0.30^{a}	48.39±4.26^{ab}
Year	2020	0.49±0.05	4.25±0.28^{b}	2.13±0.21^{a}	2.46±0.16^{a}	2.21±0.14	0.45±0.03^{b}	5.70±0.30^{a}	11.98±0.69^{a}	10.19±0.19^{b}	2.17±0.19^{b}	1.73±0.13^{b}	45.03±2.51
	2021	0.42±0.03	6.54±0.27^{a}	1.49±0.07^{b}	2.39±0.14^{a}	2.14±0.13	0.61±0.02^{a}	4.61±0.16^{b}	7.07±0.57^{b}	11.84±0.55^{a}	2.75±0.18^{a}	1.98±0.14^{a}	42.64±1.56
Statistical significance													
Cultivar		ns	***	***	***	***	***	***	***	***	***	***	***
Rootstock		*	***	***	***	***	***	***	***	***	***	***	***
Year		ns	***	***	ns	ns	***	***	***	***	***	*	ns
Cultivar × Rootstock		**	**	***	*	*	***	*	***	**	***	**	*

Data are presented as means ± standard errors (n=3). Different superscript letter in a same column (factor) denotes significant difference (Tukey's test, $p < 0.05$). Statistical significance: ns - not significant; * $p < 0.05$; ** $p < 0.01$; *** $p < 0.001$.

Table S3. Content of all individual and total hydroxycinnamic acids in fruits of sweet cherry cultivars on different rootstocks (mg/kg fresh weight, average 2020-2021).

Combination cultivar/rootstock		3-*p*-coumaroyl-quinic acid	4-*p*-coumaroyl-quinic acid	Dicaffeoyl-quinic acid 1	Dicaffeoyl-quinic acid 2	Caffeoyquinic acid glycoside 1	Caffeoyquinic acid glycoside 2	Caffeic acid glycoside 1	*cis*-3-caffeoyl-quinic acid	Neochlorogenic acid (*trans*3CQA)
Carmen/Mahaleb		0.0017±0.0002	0.012±0.002^{ef}	0.014±0.002^{a-c}	0.0155±0.0009	3.05±0.20^{c-e}	4.25±0.52^{de}	0.035±0.002	2.42±0.17^{c-f}	65.46±7.82 ^{cd}
Carmen/Colt		0.0015±0.0002	0.015±0.002^{d-f}	0.018±0.003^{a-c}	0.0098±0.0011	2.71±0.28^{d-e}	4.74±0.32^{b-e}	0.043±0.002	2.96±0.19^{a-f}	73.56±5.28 $^{b-d}$
Carmen/Oblacinska		0.0024±0.0004	0.012±0.001^{d-f}	0.010±0.002^{c}	0.0862±0.0012	2.77±0.28^{d-e}	4.30±0.40^{de}	0.045±0.005	3.08±0.04^{a-f}	91.55±4.68 ^{ab}
Carmen/M×M 14		0.0016±0.0002	0.015±0.002^{d-f}	0.012±0.002^{bc}	0.0951±0.0008	3.12±0.27^{b-e}	4.77±0.18^{b-e}	0.042±0.002	2.9±0.16^{b-f}	74.37±3.53 $^{b-d}$
Carmen/Gisela 5		0.0027±0.0004	0.015±0.002^{d-f}	0.016±0.004^{a-c}	0.0103±0.0012	3.71±0.14^{a-d}	6.16±0.3^{a-c}	0.061±0.007	4.22±0.48^{a}	99.29±11.51 ^{a}
Carmen/Gisela 6		0.0023±0.0002	0.014±0.004^{d-f}	0.018±0.004^{a-c}	0.0106±0.0010	3.43±0.25^{a-e}	5.99±0.30^{a-c}	0.045±0.003	3.13±0.24^{a-e}	81.10±6.56 $^{a-c}$
Kordia/Mahaleb		0.0012±0.0004	0.026±0.003^{a-e}	0.022±0.002^{a-c}	0.0074±0.0014	4.17±0.20^{a-c}	5.56±0.20^{a-d}	0.040±0.003	2.77±0.23^{b-f}	44.62 ±5.17 ^{ef}
Kordia/Colt		0.0011±0.0003	0.034±0.005^{ab}	0.024±0.006^{a-c}	0.0084±0.0024	3.94±0.50^{a-d}	6.75±1.06^{a}	0.049±0.005	3.36±0.34^{a-d}	60.68±5.60 ^{de}
Kordia/Oblacinska		0.0019±0.0005	0.036±0.009^{a}	0.028±0.008^{ab}	0.0079±0.0020	4.43±0.37^{ab}	6.13±0.43^{a-c}	0.050±0.003	3.41±0.22^{a-c}	61.96±5.23 ^{de}
Kordia/M×M 14		0.0012±0.0002	0.031±0.003^{a-c}	0.026±0.008 $^{a-c}$	0.0053±0.0012	3.28±0.31^{a-e}	5.67±0.48^{a-d}	0.030±0.005	2.05±0.34^{ef}	46.42±4.28 ^{ef}
Kordia/Gisela 5		0.0017±0.0006	0.031±0.008^{a-c}	0.029±0.007^{a}	0.0069±0.0018	4.61±0.39^{a}	6.75±0.35^{a}	0.052±0.004	3.63±0.27^{a-c}	68.31±9.49 ^{cd}
Kordia/Gisela 6		0.0015±0.0005	0.020±0.001^{b-f}	0.018±0.004^{a-c}	0.0078±0.0019	4.29±0.21^{ab}	6.26± 0.46^{ab}	0.054±0.009	3.71±0.62^{ab}	73.30±20.96 ^{cd}
Regina/Mahaleb		0.0015±0.0004	0.015±0.001^{e}f	0.027±0.006^{ab}	0.0061±0.0012	2.31±0.34^{e}	3.74 ±0.46^{e}	0.026±0.001	1.83±0.09^{f}	17.58±2.18 ^{g}
Regina/Colt		0.0013±0.0004	0.015±0.002^{ef}	0.026±0.004^{a-c}	0.0093±0.0015	2.63±0.45^{d-e}	4.76±0.81^{b-e}	0.039±0.007	2.72±0.54^{b-f}	40.16±4.71 ^{f}
Regina/Oblacinska		0.0010±0.0002	0.016±0.001^{d-f}	0.028±0.002^{ab}	0.0103±0.0021	3.80±0.46^{a-d}	5.66±0.35^{a-d}	0.048±0.004	3.33±0.28^{a-d}	46.80±6.91 ^{ef}
Regina/M×M 14		0.0022±0.0002	0.019±0.001^{c-f}	0.031±0.006^{a}	0.0101±0.0020	3.22±0.65^{b-e}	4.50±0.49^{c-e}	0.030±0.003	2.09±0.24^{d-f}	33.31±3.87 ^{fg}
Regina/Gisela 6		0.0023±0.0001	0.023± 0.002^{b-e}	0.022±0.002^{a-c}	0.0154±0.0073	2.80±0.14^{c-e}	4.56±0.39^{b-e}	0.095±0.006	2.12±0.20 $^{d-f}$	30.06±1.33 ^{g}
Cultivar	Carmen	0.0020±0.0001	0.049±0.001^{b}	0.015±0.001^{b}	0.0107±0.0006^{c}	5.04±0.19^{b}	3.13±0.11^{b}	0.045±0.002	3.12±0.15^{a}	40.3±1.7^{b}
	Kordia	0.0014±0.0002	0.030±0.002^{a}	0.024±0.002^{a}	0.0073±0.0007^{b}	6.19±0.223^{a}	4.12±0.15^{a}	0.046±0.002	3.16±0.16^{a}	44.3±3.5^{a}
	Regina	0.0017±0.0002	0.017±0.001^{ab}	0.027±0.002^{a}	0.0102±0.0016^{a}	4.64±0.25^{b}	2.95±0.21^{b}	0.048±0.013	2.42±0.16^{b}	44.1±2.6^{a}
Rootstock	Mahaleb	0.0014±0.0014	0.018±0.002	0.021±0.002	0.010±0.001	4.52±0.29^{c}	3.18±0.23	0.034±0.002	2.34±0.13^{c}	33.4±2.7^{c}
	Colt	0.0013±0.0001	0.021±0.003	0.023±0.003	0.009±0.001	5.42±0.49^{bc}	3.10±0.27	0.044±0.003	3.01±0.22^{ab}	45.4±5.8^{ab}
	Oblacinska	0.0018±0.0003	0.021±0.004	0.022±0.003	0.009±0.001	5.36±0.28^{bc}	3.67±0.27	0.048±0.003	3.28±0.17^{ab}	48.3±3.9^{a}
	MxM 14	0.0017±0.0001	0.022±0.002	0.023±0.004	0.008±0.001	4.98±0.25^{bc}	3.21±0.24	0.034±0.002	2.35±0.17^{c}	41.2±4.3^{bc}
	Gisela 5	0.0022±0.0004	0.023±0.002	0.023±0.002	0.009±0.001	6.45±0.26^{a}	4.16±0.24	0.057±0.004	3.93±0.28^{a}	46.5±6.0^{ab}
	Gisela 6	0.0020±0.0004	0.018±0.002	0.020±0.002	0.011±0.002	5.61±0.28^{ab}	3.51±0.19	0.065 ±0.021	2.99±0.27^{ab}	41.0±4.3^{bc}
Year	2020	0.0017±0.0002	0.022±0.002	0.024±0.002^{a}	0.009±0.0001	5.19±0.25	3.19±0.16^{b}	0.045±0.002	2.76±0.15^{b}	43.8±2.5
	2021	0.0017±0.0001	0.019±0.001	0.019±0.002^{b}	0.010±0.0001	5.47±0.14	3.67±0.12^{a}	0.046±0.002	3.10±0.12^{a}	41.8±1.6
Statistical significance										
Cultivar		ns	***	***	*	***	***	ns	***	***
Rootstock		ns	***	ns	ns	ns	**	ns	***	***
Year		ns	ns	*	ns	***	ns	ns	***	***
Cultivar × Rootstock		ns	*	ns	ns	**	*	ns	*	**

Data are presented as means ± standard errors (n=3). Different superscript letter in a same column (factor) denotes significant difference (Tukey's test, $p < 0.05$). Statistical significance: ns - not significant; * $p < 0.05$; ** $p < 0.01$; *** $p < 0.001$.

Table S3. Continued

**Caffeic acid hexoside 1**	**Caffeoylquinic acid glycoside 3**	**Caffeic acid hexoside 2**	***p*-coumaric acid hexoside**	***trans*-3-*p*-coumaroyl-quinic acid**	***trans* 4-caffeoyl-quinic acid**	***trans* 5-caffeoyl-quinic acid**	***cis*-3-*p*-coumaroyl-quinic acid**	***cis* 3-feruloyl-quinic acid**	***cis* 4-caffeoyl-quinic acid**
0.08±0.01^{de}	10.97±1.89	29.33±4.91^{f-i}	5.48±0.92^{e-h}	29.67±3.61^{fg}	1.12±0.14^{d-f}	7.59±0.99	0.13±0.02	0.06±0.01	0.51±0.05
0.09±0.00^{de}	17.66±1.56	51.71±5.44^{b-g}	9.66±1.02^{c-e}	32.39±2.72^{ef}	1.22±0.10^{d-f}	9.51±0.44	0.17±0.01	0.07±0.01	0.60±0.04
0.12±0.03^{a-e}	13.34±1.42	36.42±3.17^{e-i}	6.81±1.71^{d-h}	56.65±3.55^{a}	2.14±0.13^{a}	11.19±0.58	0.19±0.01	0.08±0.01	0.65± 0.05
0.11±0.00^{b-e}	18.49±1.33	49.70±5.10^{b-h}	9.29±1.29^{c-f}	37.05±3.0 ^{de}	1.40±0.12^{c-e}	8.87±0.42	0.15±0.01	0.07±0.01	0.60±0.06
0.14±0.02^{a-d}	17.70±1.13	42.08±4.66^{d-i}	7.87±0.81^{d-g}	50.59±4.95^{ab}	1.91±0.30^{ab}	10.84±1.03	0.19±0.02	0.08±0.01	0.71±0.05
0.08±0.01^{de}	9.82±1.81	28.70±2.55^{g-i}	5.36±0.85^{f-h}	39.45±3.78^{cd}	1.49±0.14^{b-d}	9.18±0.84	0.16±0.01	0.07±0.01	0.63±0.06
0.11±0.02^{b-e}	16.04±0.52	45.69±3.64^{b-i}	8.54±0.68^{c-f}	25.61±1.41^{gh}	0.97±0.05^{e-f}	6.90±0.26	0.12±0.01	0.05±0.01	0.35±0.02
0.17±0.02^{a}	24.80±1.52	85.71±7.56^{a}	16.02±2.03^{a}	30.23±3.39^{fg}	1.14±0.13^{d-f}	12.93±1.74	0.22±0.05	0.10±0.02	0.48±0.03
0.15±0.01^{a-c}	19.74±1.98	57.24±5.48^{b-f}	10.70±1.96^{b-d}	37.06±3.01^{de}	1.40±0.11^{c-e}	10.65±1.05	0.19±0.02	0.08±0.01	0.44±0.02
0.16±0.03^{ab}	27.89±2.81	76.62±6.80^{ab}	14.32±1.83^{ab}	27.84±2.50^{fg}	1.05±0.09^{d-f}	8.23±1.37	0.14±0.02	0.06±0.01	0.38±0.02
0.13±0.01^{a-d}	19.46±1.28	57.62±5.88^{a-e}	10.77±1.41^{b-d}	45.30±4.05^{bc}	1.71±0.15^{a-c}	12.21±0.59	0.21±0.01	0.09±0.01	0.50±0.02
0.10±0.02^{c-e}	14.18±0.94	37.12±3.78^{e-i}	6.94±1.08^{d-h}	45.65±5.49^{b}	1.73±0.19^{a-c}	11.34±1.24	0.20±0.04	0.08±0.02	0.52±0.05
0.06±0.01^{e}	9.06±0.89	18.20±1.77^{i}	3.40±0.33^{h}	8.61±1.47^{h}	0.33±0.05^{g}	2.54±0.26	0.04±0.01	0.02±0.01	0.39±0.02
0.10±0.02^{c-e}	13.05±2.82	43.39±4.20^{b-i}	8.11±1.72^{d-g}	21.20±2.85^{hi}	0.80±0.11^{fg}	6.07±1.25	0.11±0.02	0.05±0.01	0.35±0.03
0.13±0.02a^{-d}	16.04±1.51	66.48±5.66^{a-d}	12.43±1.18^{a-c}	25.19±4.69^{gh}	0.95±0.18 ^{ef}	8.16±1.18	0.14±0.02	0.06±0.01	0.37±0.04
0.09±0.01^{de}	14.75±1.58	67.75±7.80^{a-c}	12.66±1.70^{ab}	19.73 ±3.90^{hi}	0.75±0.15^{fg}	5.69±1.34	0.10±0.02	0.04±0.01	0.40±0.03
0.11±0.01^{a-e}	14.31±1.06	23.07±2.90^{hi}	4.00±0.50^{gh}	16.60±1.77^{ij}	0.82±0.10^{fg}	5.15±0.70	0.20±0.01	0.09±0.02	0.45±0.04
0.11±0.01^{b}	14.66±1.27^{b}	39.66±2.99^{b}	7.41±0.56^{b}	40.97±2.35^{a}	1.55±0.09^{a}	9.53±0.35 ^{a}	0.17±0.01^{a}	0.07±0.02^{a}	0.62±0.04^{a}
0.14±0.01^{a}	20.35±1.37^{a}	60.00±5.39^{a}	11.21±1.01^{a}	35.28±2.35^{b}	1.33±0.09^{b}	10.38±0.71 ^{a}	0.18±0.01^{a}	0.08±0.1^{a}	0.44±0.02^{b}
0.10±0.01^{b}	13.44±1.34^{b}	43.78±4.04^{b}	8.12±1.14^{b}	18.26±1.68^{c}	0.73±0.09^{c}	5.52±0.55 ^{b}	0.12±0.02^{b}	0.05±0.01^{b}	0.39±0.02^{b}
0.09±0.01^{c}	12.02±1.31^{c}	31.07±3.39^{c}	5.81±0.63^{c}	21.30±2.56^{d}	0.80±0.10^{d}	5.67±0.64 ^{d}	0.10±0.01^{b}	0.04±0.01^{b}	0.41±0.02^{c}
0.12±0.01^{ab}	18.50±1.34^{a}	60.27±5.75^{ab}	11.26±1.63^{ab}	27.94±2.01^{cd}	1.06±0.08^{c}	9.50±1.17 ^{bc}	0.17±0.02^{a}	0.07±0.01^{a}	0.48±0.03^{a-c}
0.13±0.01^{a}	16.38±1.78^{b}	53.38±4.45^{ab}	9.98±1.21^{ab}	39.63±3.76^{ab}	1.50±0.14 ^{b}	10.00±0.62 ^{ab}	0.17±0.01^{a}	0.07±0.01^{a}	0.48±0.04^{a-c}
0.12±0.01^{ab}	20.38±2.08^{a}	64.69±5.60^{a}	12.09±1.42^{a}	28.21±2.45^{c}	1.07±0.09^{c}	7.60±0.70 ^{cd}	0.13±0.01^{ab}	0.06±0.01^{ab}	0.46±0.03^{bc}
0.13±0.01^{a}	18.58±1.85^{a}	49.85±4.03^{b}	9.32±1.50 ^{b}	47.94±4.33^{a}	1.81±0.16^{a}	11.52±0.60 ^{a}	0.20±0.01^{a}	0.09±0.01^{a}	0.60±0.06^{a}
0.10±0.01^{bc}	12.77±1.04^{bc}	29.63±2.85^{c}	5.43±0.54 ^{c}	33.90±4.66^{bc}	1.34±0.16^{bc}	8.56±1.00 ^{bc}	0.19±0.03^{a}	0.08±0.02^{a}	0.53±0.05^{ab}
0.09±0.01^{b}	14.70±1.41^{b}	56.52±5.25^{a}	10.53±0.99^{a}	29.73±2.06^{b}	1.15±0.08^{b}	8.77±0.57	0.17±0.01	0.07±0.01	0.55±0.03^{a}
0.14±0.01^{a}	17.92±0.83^{a}	39.58±2.00^{b}	7.40±0.37^{b}	34.83±0.33^{a}	1.32±0.09^{a}	8.53±0.51	0.15±0.01	0.06±0.01	0.43±0.02^{b}
***	***	***	***	***	***	***	*	*	***
***	***	***	***	***	***	***	**	**	**
***	**	***	***	***	***	ns	ns	ns	***
**	ns	***	***	***	***	***	ns	ns	ns

Table S3. Continued

trans 3-feruloyl-quinic acid	Sinapic acid hexoside	*trans*-5-feruloyl-quinic acid	Caffeic acid glycoside 2	Feruloyl hexoside	*cis* 5-caffeoyl-quinic acid	*trans*-4-*p*-coumaroylquinic acid	*cis*-4-*p*-coumaroylquinic acid	5-*p*-coumaroyl-quinic acid	Total hydroxy-cinnamic acids
0.69±0.07^{b-e}	0.21±0.02^{b-d}	0.30±0.03^{ef}	0.11±0.02^{cd}	0.32±0.03^{de}	2.57±0.12^{e-g}	0.69±0.09^{c-e}	0.31±0.02^{c}	0.19±0.02	111.1±14.8^{fg}
0.83±0.05^{a-c}	0.25±0.02^{ab}	0.37±0.04^{d-f}	0.14±0.01^{b-d}	0.39±0.04^{c-e}	2.29±0.23^{d}	0.48±0.04^{de}	0.35±0.02^{c}	0.25±0.03	156.7±19.5^{b-d}
0.89±0.08^{ab}	0.27±0.03^{ab}	0.32±0.03^{ef}	0.12±0.03^{cd}	0.34±0.04^{de}	2.37±0.20^{fd}	0.92±0.06^{a-c}	0.49±0.05^{c}	0.30±0.03	167.2±21.7^{ab}
0.82±0.09^{a-c}	0.25±0.03^{a-c}	0.43±0.04^{a-e}	0.16±0.02^{a-c}	0.46±0.06^{a-e}	2.50±0.21^{e-g}	0.71±0.12^{c-e}	0.38±0.02^{c}	0.26±0.04	161,1±19.8^{a-c}
0.97±0.10^{a}	0.29±0.03^{a}	0.40±0.04^{b-f}	0.15±0.03^{a-d}	0.42±0.04^{a-e}	3.00±0.19^{c-g}	0.90±0.19^{a-c}	0.45±0.04^{c}	0.28±0.03	190.9±26.8^{a}
0.87±0.08^{ab}	0.26±0.02^{ab}	0.38±0.03 $^{c-f}$	0.14±0.02^{b-d}	0.40±0.05^{b-e}	2.80±0.22^{c-g}	0.76±0.17^{c-e}	0.35±0.03^{c}	0.20±0.04	123.6±14.1^{de}
0.47±0.03^{fg}	0.14±0.01^{g}	0.45±0.02^{a-e}	0.17±0.01^{a-c}	0.47±0.05^{a-e}	3.10±0.10^{b-f}	0.75±0.11^{c-e}	0.85±0.04^{b}	0.23±0.02	139.6±15.0^{c-e}
0.66±0.04^{c-f}	0.20±0.01^{c-f}	0.57±0.05 ^{a}	0.22±0.03^{a}	0.60±0.06^{ab}	3.58±0.16^{a-c}	0.71±0.12^{c-e}	0.83±0.08^{b}	0.32±0.04	198.4±17.8^{a}
0.60±0.02^{d-g}	0.18±0.01^{d-g}	0.55±0.05^{ab}	0.21±0.02^{a}	0.58±0.05^{a-c}	3.44±0.26^{a-d}	0.98±0.13^{a-c}	0.81±0.07^{b}	0.30±0.03	179.1±19.9^{ab}
0.52±0.03^{d-g}	0.16±0.01^{d-g}	0.54±0.05^{a-c}	0.20±0.01^{a}	0.57±0.05^{a-c}	3.01±0.17^{c-g}	0.80±0.13^{b-d}	0.79±0.05^{b}	0.28±0.03	187.5±25.8^{ab}
0.69±0.03^{b-e}	0.21±0.01^{c-e}	0.53±0.02^{a-d}	0.20±0.01^{a}	0.56±0.02^{a-c}	3.90±0.27^{a}	1.11±0.18^{ab}	0.96±0.03^{b}	0.40±0.02	191.1±22.4^{a}
0.71±0.07^{b-d}	0.21±0.03^{c-f}	0.51±0.06^{a-d}	0.19±0.02^{ab}	0.54±0.06^{a-c}	3.85±0.32^{ab}	1.20±0.15^{a}	0.94±0.05^{b}	0.34±0.05	154.4±18.7^{b-d}
0.54±0.03^{d-g}	0.16±0.01^{e-g}	0.26±0.04^{f}	0.10±0.01^{d}	0.27±0.04^{e}	3.42±0.17^{a-d}	0.42±0.04^{e}	0.84±0.08^{b}	0.23±0.04	75.9±13.2^{g}
0.48±0.02^{fg}	0.14±0.01^{g}	0.43±0.04 $^{a-e}$	0.16±0.03^{a-c}	0.46±0.05^{a-e}	2.65±0.22^{d-g}	0.74±0.12^{c-e}	0.94±0.04^{b}	0.27±0.03	122.8±15.9^{ef}
0.51±0.05^{e-g}	0.15±0.02^{fg}	0.58±0.05^{a}	0.22±0.02^{a}	0.61±0.07^{a}	3.24±0.11^{a-e}	0.94±0.16^{a-c}	1.35±0.11^{a}	0.27±0.05	156.8±26.1^{b-d}
0.55±0.04^{d-g}	0.16±0.01^{e-g}	0.47±0.04^{a-e}	0.17±0.02^{a-c}	0.49±0.05^{a-d}	3.23±0.16^{a-e}	0.76±0.14^{c-e}	1.23±0.14^{ab}	0.25±0.03	153..9±23.2^{b-e}
0.41±0.07^{g}	0.16±0.03^{d-g}	0.51±0.03^{a-d}	0.17±0.01^{a-c}	0.57±0.05^{a-c}	3.09±0.08^{b-f}	0.90±0.08^{a-c}	1.33±0.06^{a}	0.52±0.06	91.4±24.4^{fg}
0.85±0.05^{a}	0.25±0.01	0.37±0.03^{c}	0.14±0.01^{b}	0.39±0.03^{c}	2.59±0.09 ^{c}	0.74±0.07^{b}	0.39±0.02^{c}	0.24±0.02	163.4±9.9^{a}
0.61±0.03^{b}	0.18±0.01	0.53±0.02^{a}	0.20±0.01^{a}	0.56±0.02^{a}	3.48±0.11 ^{a}	0.93±0.10^{a}	0.86±0.08^{b}	0.31±0.02	154.5±15.0^{a}
0.49±0.02^{c}	0.16±0.01	0.45±0.03^{b}	0.16±0.02^{b}	0.48±0.03^{b}	3.13 ± 0.08 ^{b}	0.75±0.08^{b}	1.14±0.07^{a}	0.31±0.04	139.9±13.5^{b}
0.57±0.03^{c}	0.17±0.01^{c}	0.34±0.03^{b}	0.13±0.01^{b}	0.35±0.03^{b}	3.03 ± 0.11^{bc}	0.62±0.08^{c}	0.67±0.07^{b}	0.21±0.02^{b}	105.5±17.4^{c}
0.66±0.04^{b}	0.20±0.01^{b}	0.46±0.04^{a}	0.17±0.02^{a}	0.48±0.04^{a}	2.84 ± 0.17^{c}	0.65±0.09^{c}	0.71±0.08^{b}	0.28±0.03^{ab}	155.9±24.2^{b}
0.66±0.06^{b}	0.20±0.02^{b}	0.48±0.05^{a}	0.18±0.02^{a}	0.51±0.05^{a}	3.02±0.16^{bc}	0.94±0.09^{a}	0.88±0.11^{a}	0.29±0.03^{ab}	163.64±17.2^{ab}
0.63±0.05^{bc}	0.19±0.01^{bc}	0.48±0.05^{a}	0.18±0.01^{a}	0.51±0.03^{a}	2.91±0.13^{bc}	0.76±0.09^{b}	0.80±0.10^{ab}	0.26±0.02^{ab}	172.49±21.8^{b}
0.83±0.09^{a}	0.25±0.03^{a}	0.47±0.05^{a}	0.18±0.02^{a}	0.49±0.05^{a}	3.45±0.21^{a}	1.01±0.11^{a}	0.71±0.08^{b}	0.34±0.03^{ab}	180.97±22.0^{a}
0.66±0.08^{b}	0.21±0.02^{b}	0.47±0.04^{a}	0.17±0.02^{a}	0.50±0.05^{a}	3.25± .18^{ab}	0.95±0.16^{a}	0.87±0.10^{a}	0.35±0.03^{a}	124.98±21.5^{c}
0.73±0.04^{a}	0.22±0.01^{a}	0.38±0.03^{b}	0.14±0.01^{b}	0.41±0.03^{b}	2.99±0.07	0.41±0.02^{b}	0.71±0.04^{b}	0.29±0.03	149.5±11.1 ^{b}
0.59±0.02^{b}	0.18±0.01^{b}	0.51±0.01^{a}	0.19±0.01^{a}	0.54±0.02^{a}	3.13±0.11	1.21±0.07^{a}	0.85±0.06^{a}	0.29±0.01	153.1±10.1^{a}
***	***	***	***	***	***	***	***	ns	***
*	**	***	***	***	*	***	***	*	***
***	***	***	***	***	ns	***	***	ns	***
***	*	***			**	**	***	ns	*

Table S4. Meteorological conditions during the first six months in the years of study (2020-2021).

Year	Month	Average temperature (°C)	Precipitation sum (mm)	Insolation (hours)
2020	January	2.0	22.1	122.7
	February	7.6	55.9	120.2
	March	9.1	48.0	149.4
	April	14.3	8.9	293.1
	May	16.6	70.9	212.6
	June	20.9	158.5	229.6
2021	January	2.8	68.6	76.6
	February	6.5	34.4	130.4
	March	7.2	49.3	179.1
	April	10.6	50.7	187.9
	May	17.4	93.4	264.6
	June	24.3	34.2	326.3
